# ASCL1 regulates and cooperates with FOXA2 to drive terminal neuroendocrine phenotype in prostate cancer

**DOI:** 10.1172/jci.insight.185952

**Published:** 2024-12-06

**Authors:** Shaghayegh Nouruzi, Takeshi Namekawa, Nakisa Tabrizian, Maxim Kobelev, Olena Sivak, Joshua M Scurll, Cassandra Jingjing Cui, Dwaipayan Ganguli, Amina Zoubeidi

**Affiliations:** 1 Department of Urologic Sciences, The University of British Columbia, Vancouver, British Columbia, Canada.; 2 Vancouver Prostate Centre, Vancouver, British Columbia, Canada.

**Keywords:** Cell biology, Epigenetics, Neuroendocrine regulation, Prostate cancer

## Abstract

Lineage plasticity mediates resistance to androgen receptor pathway inhibitors (ARPIs) and progression from adenocarcinoma to neuroendocrine prostate cancer (NEPC), a highly aggressive and poorly understood subtype. Neuronal transcription factor ASCL1 has emerged as a central regulator of the lineage plasticity driving neuroendocrine differentiation. Here, we showed that ASCL1 was reprogrammed in ARPI-induced transition to terminal NEPC and identified that the ASCL1 binding pattern tailored the expression of lineage-determinant transcription factor combinations that underlie discrete terminal NEPC identity. Notably, we identified FOXA2 as a major cofactor of ASCL1 in terminal NEPC, which is highly expressed in ASCL1-driven NEPC. Mechanistically, FOXA2 and ASCL1 interacted and worked in concert to orchestrate terminal neuronal differentiation. We identified that prospero homeobox 1 was a target of ASCL1 and FOXA2. Targeting prospero homeobox 1 abrogated neuroendocrine characteristics and led to a decrease in cell proliferation in vitro and tumor growth in vivo. Our findings provide insights into the molecular conduit underlying the interplay between different lineage-determinant transcription factors to support the neuroendocrine identity and nominate prospero homeobox 1 as a potential target in ASCL1-high NEPC.

## Abstract 2

ASCL1 mediate terminal neuroendocrine differentiation of prostate cancer by regulating lineage-determinant transcription factor FOXA2. We identified that Prospero-related homeobox 1 is a target of ASCL1 and FOXA2 and nominated it as a potential therapeutic target in ASCL1-high NEPC.

## Graphical abstract

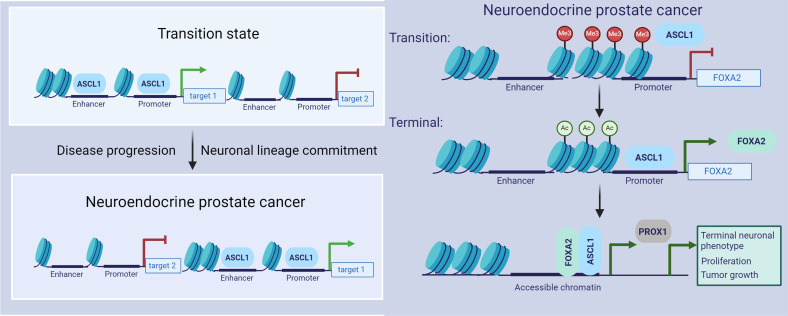

## Introduction

Neuroendocrine prostate cancer (NEPC) is aggressive, poorly differentiated, and characterized by the expression of neuroendocrine markers (1–3). De novo NEPC is rare, accounting for 1%–2% of diagnosed cases (4, 5), while treatment-induced NEPC accounts for approximately 20% of all late-stage prostate cancer cases (6–10). It is on the rise as a resistance mechanism triggered by potent androgen receptor pathway inhibitors (ARPIs) (11, 12) such as enzalutamide (ENZ). NEPC is independent of androgen receptor (AR) signaling, and the standard of care is platinum-based chemotherapy, which provides modest improvement in overall survival (13, 14). The lack of curative therapies for NEPC stems from our limited understanding of the mechanism driving the development of this aggressive phenotype. The urgent need for effective diagnostic markers and therapeutic targets is highlighted by the aggressive nature and poor prognosis. Furthermore, as resistance to ARPIs becomes more prevalent, understanding the mechanisms behind NEPC’s resistance becomes more critical.

The transcriptional landscape of NEPC is complex, governed by key regulatory factors that define distinct subtypes. Recent reports indicate that a limited number of transcription factors (TFs) collaboratively exert control over the cell’s lineage identity by regulating NE gene expression programs (15). These lineage-specific TFs are pivotal in driving the subtypes observed in NEPC (16–18), mirroring small cell lung cancer (SCLC) (19). Both SCLC and NEPC share common characteristics, such as small cell phenotype, disease aggressiveness, and dependence on key neuroendocrine TFs, including ASCL1 (16, 20). Exploring the parallels between NEPC and SCLC may offer insights into the mechanisms of resistance and potential therapeutic vulnerabilities. This study focuses on unraveling the transcriptional regulatory networks driving NEPC, with a particular emphasis on its similarities to SCLC.

ASCL1 is part of a core regulatory circuit of developmental regulators that promotes oncogenicity in neuroblastoma (21–23). It modulates both the proliferation and differentiation of neurons (24), with elevated levels leading to the termination of the cell cycle and triggering differentiation processes (25, 26). In NEPC, a subset of patients can be identified by high levels of ASCL1 (16, 27). ASCL1 is a critical determinant of neuroendocrine lineage identity and is required for initiating neuroendocrine differentiation (16). ASCL1 expression and activity increase after ARPIs and continue to rise as the disease progresses, reaching an apex in NEPC (16, 28). This suggests that ASCL1 may have lineage-specific targets and distinct transcriptional signatures at different stages of the disease (lineage plasticity, cell fate decision-making, and lineage maturation). At each stage, ASCL1 might have specialized functions activating distinct sets of target genes. However, ASCL1’s transcriptome in terminal NEPC has not yet been fully characterized.

In this study, we investigated ASCL1’s cistrome and transcriptome in a series of terminally differentiated patient-derived xenografts (PDXs) and cell lines and identified ASCL1 core-regulated targets. We demonstrated that FOXA2 was specifically expressed at the terminal stage of NEPC and where it was acetylated. We found that ASCL1 and FOXA2 cobound in accessible regions and regulated genes required for neuronal identity such as prospero homeobox 1 (PROX1). PROX1 is an oncogene in SCLC (29) and plays a key role in neurogenesis (30). It influences colon cancer progression (31) and tumor metastasis (32) and supports neuroblasts (33). We found that PROX1 regulated cell proliferation and tumor growth in NEPC. Our data shed light on the transcriptional dynamics facilitated by ASCL1 and FOXA2 and identified PROX1 as a potential target in NEPC.

## Results

### ASCL1 regulates pro-neuronal factors in NEPC.

**Figure 1:**
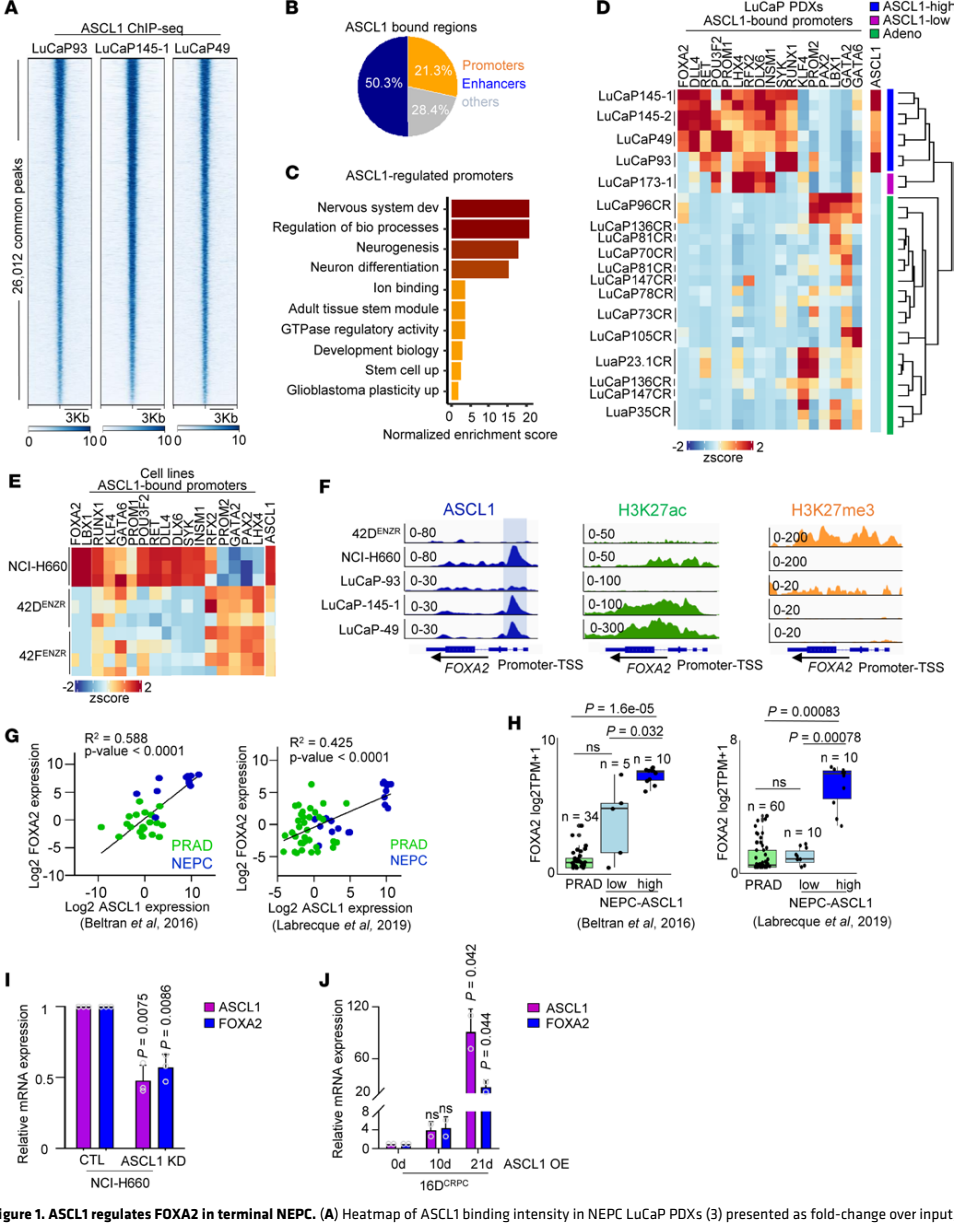
ASCL1 regulates FOXA2 in terminal NEPC. (**A**) Heatmap of ASCL1 binding intensity in NEPC LuCaP PDXs (3) presented as fold-change over input, with each horizontal line representing a 3 kb locus. (**B**) Genomic annotation is shown as a percentage of all peaks. Introns and intergenic regions were called enhancers, and UTRs and transcription start sites (TSSs) were called others. (**C**) Pathways associated with ASCL1-bound promoters are shown as normalized enrichment scores with pathways *P* < 0.05; statistical analysis was calculated by gProfiler. (**D**) The heatmap shows the expression of ASCL1-bound promoters in LuCaP PDXs. Samples are clustered based on ASCL1 expression (*n* = 18). (**E**) Heatmap shows expression of ASCL1-bound promoters in 42D^ENZR^, 42F^ENZR^, and de novo NEPC cell line NCI-H660. (**F**) Visualization of genomic loci of FOXA2 using IGV showing relative occupancy of ASCL1 over input (left panel), the density of acetylation at H3K27 (H3K27ac) (middle panel), and H3K27 trimethylation (H3K27me3) (right panel) in 42D^ENZR^, NCI-H660, and ASCL1-driven PDXs. (**G**) The correlation between FOXA2 and ASCL1 expression (log_2_ transcripts per million, log_2_TPM) in the patient datasets (3, 9), with each dot representing a patient tumor. Significance was evaluated by linear regression *t* test. (**H**) FOXA2 gene expression in PRAD, ASCL1-low NEPC, and ASCL1-high NEPC populations in the patient datasets (3, 9), with significance assessed using 2-tailed unpaired *t* test. Bonferroni’s correction was applied to adjust for multiple comparisons. Jittered points represent individual samples. (**I**) Relative mRNA expression of ASCL1 and FOXA2 in NCI-H660 following knockdown (KD) of ASCL1 using shRNA. Data are reported relative to nontransfected cells (CTL) (mean ± SD; *n* = 3). (**J**) Relative mRNA expression of ASCL1 and FOXA2 in 16D^CRPC^ following doxycycline (Dox) induction of ASCL1. Data are reported relative to day 0 (mean ± SD, *n* = 2). OE, overexpression.

To elucidate ASCL1’s role in NEPC, we used ASCL1 ChIP-Seq in LuCaP PDXs (18) and outlined the ASCL1 binding landscape. We identified approximately 26,000 shared peaks across 3 PDX samples (Figure 1A), mapped to approximately 15,000 genes (Supplemental Figure 1A; supplemental material available online with this article; https://doi.org/10.1172/jci.insight.185952DS1). Notably, 21% of ASCL1-bound sites were located at promoters (Figure 1B), regulating key pathways of neuronal differentiation (Figure 1C). Next, we investigated ASCL1’s direct interaction with promoters. Integrating RNA-Seq data, we explored the expression of genes with ASCL1-bound promoters in ASCL1-high and ASCL1-low NEPC, as well as prostate adenocarcinoma (PRAD) in LuCaP PDXs. We observed differences in the gene expression (Supplemental Figure 1B and Figure 1D) and showed that ASCL1 has a unique transcriptomic profile in the ASCL1-high phenotype. ASCL1-bound promoters belonging to terminal neuronal phenotypes, such as FOXA2 (34), LHX4 (35), INSM1 (36, 37), and DLX6 (38) (Figure 1D). A similar pattern of expression was observed comparing the de novo NEPC model NCI-H660 with treatment-induced (tNEPC) models 42D^ENZR^ and 42F^ENZR^ (Figure 1E). We observed that ASCL1 has different binding patterns in the tNEPC compared with the terminal NEPC (Supplemental Figure 1C). To identify ASCL1-regulated genes in a lineage-specific manner, we compared the expression of ASCL1-bound promoters between NCI-H660 and 42D^ENZR^. Ranking TFs enriched in one phenotype versus the other, we identified FOXA2 to be specifically upregulated in NCI-H660 compared with 42D^ENZR^ cells (Supplemental Figure 1D), supporting the notion that ASCL1 has lineage-specific targets in tNEPC compared with terminal NEPC.

FOXA2 has been reported to be upregulated in NEPC (34, 39) and small cell neuroendocrine carcinomas (34, 40). However, the mechanisms by which it is regulated in neuroendocrine tumors remain unknown. We observed that ASCL1 bound to the FOXA2 promoter (Figure 1F), which was highly acetylated at H3K27 (a mark of active transcription) in LuCaP and NCI-H660 (Figure 1F), correlating with FOXA2 expression (Figure 1, D and E). We noted that FOXA2 was not expressed in LuCaP 93 (an ASCL1-high NEPC PDX), which exhibited low ASCL1 binding affinity to the FOXA2 promoter (Figure 1D). This was in alignment with an absence of acetylation and high levels of H3K27me3 (a mark for transcription repression) at the FOXA2 promoter and gene body (Figure 1F). Interestingly, tNEPC 42D^ENZR^ exhibited a similar pattern as LuCaP 93 (Figure 1F). Of note, AR did not bind to the FOXA2 promoter (Supplemental Figure 1E), and FOXA2 expression was not induced by ARPI treatment in cell lines (Supplemental Figure 1, F and G) or patients (41) (Supplemental Figure 1H). These data suggest that despite the expression of ASCL1, its binding pattern is a key determinant of gene expression.

To further investigate the link between ASCL1 and FOXA2, we examined the expression of FOXA2 and its correlation with ASCL1 in a cohort of patients with PRAD and NEPC (3, 9). We observed a significant positive correlation between ASCL1 and FOXA2 expression in patient datasets — *R*^2^ = 0.58 in Beltran et al. (9) and *R*^2^ = 0.42 in Labrecque et al. (3), *P* < 0.05 — (Figure 1G), with FOXA2 exclusively upregulated in ASCL1-high NEPC subtypes (Figure 1H). Although it has been reported that ASCL1 reprograms the FOXA1 cistrome (42), we did not observe any correlation between FOXA1 and ASCL1 expression (Supplemental Figure 1I), highlighting a tight link between ASCL1 and FOXA2.

Given that ASCL1 binds to the FOXA2 promoter, we focused on whether ASCL1 is a direct regulator of FOXA2. Indeed, the loss of ASCL1 resulted in downregulation of FOXA2 expression alongside other neuroendocrine markers in NCI-H660 (Figure 1I). To validate the regulatory role of ASCL1 on FOXA2 expression, we investigated the expression of FOXA2 in castration-resistant prostate cancer (CRPC) cell line (16D^CRPC^) overexpressing ASCL1 as well as in an established in vitro NEPC model of C4-2B containing functional loss of *TP53*, *RB1*, and overexpression of *MYCN* and *BCL2* (PRMB) (National Center for Biotechnology [NCBI] Gene Expression Omnibus [GEO] GSE225013). Overexpression of ASCL1 in both models elevated FOXA2 levels (Figure 1J and Supplemental Figure 1J). These results indicate that ASCL1 acts upstream of FOXA2 to govern its expression in NEPC, a factor that can further potentiate neuronal characteristics. Furthermore, ASCL1 binds upstream of the FOXA2 promoter in SCLC, and knocking down ASCL1 leads to FOXA2 downregulation (20), consistent with our observations in NEPC.

### The FOXA2 cistrome is conserved in NEPC and SCLC.

FOXA1 has been well studied in various stages of prostate cancer. It is known to cooperate with the AR to maintain the luminal epithelial lineage in CRPC (43), while it is reprogrammed by ASCL1 to neuroendocrine regulatory regions in NEPC (42). FOXA1 has similar expression in all Gleason grades of prostate carcinomas while FOXA2 has been found in some prostate cancer with high Gleason scores and in neuroendocrine small cell carcinomas (44). The shift from FOXA1 to FOXA2 expression represents a notable marker of lineage plasticity and disease progression within prostate tumors, highlighting a transition toward a more aggressive phenotype. Previous studies in a mouse model driven by the loss of tumor-suppressive genes (*PTEN*, *TP53*, and *RB1*) reported that FOXA2 plays a key role in the progression of NEPC (39), and in a genetically engineered TRAMP mouse model, FOXA2 expression was uniformly identified in synaptophysin-positive (SYP-positive) tumors (45). However, the role of FOXA2 in NEPC remains to be explored.

**Figure 2:**
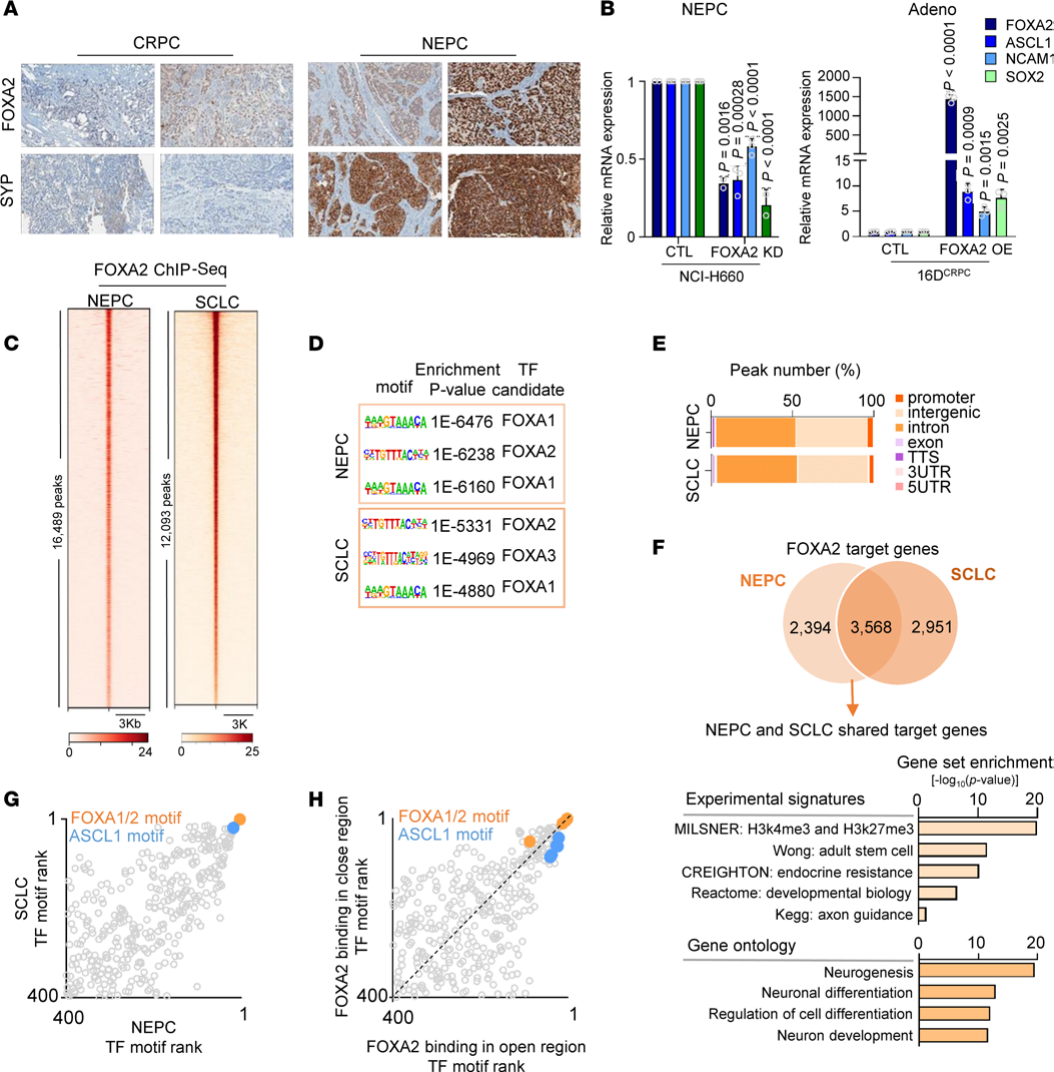
FOXA2 in NEPC. (**A**) Immunohistochemical staining for FOXA2 and SYP in CRPC and NEPC clinical samples. Original magnification, ×20. (**B**) Relative mRNA expression of neuronal and stem cell genes normalized to GAPDH in NCI-H660 following FOXA2 KD using shRNA (left) and 16D^CRPC^ (CTL) following OE of FOXA2 (right). Data are reported relative to nontransfected cells (mean ± SD, *n* = 3 biologically independent samples). Statistical analysis was measured using a 2-tailed unpaired *t* test. Adeno, adenocarcinoma. (**C**) Heatmap of FOXA2 binding intensity as fold-change over input, with each horizontal line representing a 3 kb locus. (**D**) Motif enrichment analysis from FOXA2 ChIP-Seq in NEPC (top panel) and SCLC (bottom panel) shows the top 3 motifs, called using HOMER. (**E**) Genomic annotation of FOXA2 binding location in NEPC and SCLC cell models presented as the percentage of total peaks. (**F**) Venn diagram showing the overlap of FOXA2 target genes in NEPC and SCLC cell models (upper panel); pathways associated with FOXA2 shared target genes in NEPC and SCLC shown as negative log_10_ of *P* value, with *P* < 0.05 (lower panel). KEGG, Kyoto Encyclopedia of Genes and Genomes. (**G**) Using FOXA2 ChIP-Seq, the motifs enriched in NEPC and SCLC were compared. Motifs were ranked based on *P* value, with rank number 1 showing the most enriched. (**H**) Comparing the motifs enriched in FOXA2-bound open or closed chromatin. Motifs were ranked based on *P* value, with rank number 1 showing the most enriched.

First, we observed an increase in FOXA2 expression in NEPC compared with CRPC patient tumors (3, 9, 46) (Figure 1H and Supplemental Figure 2A), LuCaP PDXs (3) (Supplemental Figure 2B), and SCLC compared with non-SCLC (NSCLC) samples (47) (Supplemental Figure 2C). Notably, increased FOXA2 expression in NEPC positively correlated with increased neuroendocrine score (while FOXA1 correlated with AR score) in patient datasets (3, 9, 46) (Supplemental Figure 2D). In alignment, FOXA2 expression was elevated in NEPC compared with lineage-plastic neuroendocrine-like or adenocarcinoma cell lines (Supplemental Figure 2E). Using immunohistochemistry (IHC), we verified the increased expression of FOXA2 in a subset of NEPC (Figure 2A and Supplemental Figure 2F).

The knockdown of FOXA2 in the NEPC cell line reduced the expression of plasticity genes, while its overexpression in CRPC was sufficient to induce the expression of these genes (Figure 2B). To establish the functional role of FOXA2 in NEPC and SCLC, we mapped FOXA2 occupancy in NEPC and SCLC cell models (NCI-H660 and NCI-H889, respectively) (Figure 2C). Validating the ChIP-Seq, we identified top enriched motifs belonging to the FOXA family (Figure 2D). The majority of FOXA2 binding sites corresponded to enhancer regions (intron and intergenic) (Figure 2E). To identify FOXA2 core target sites, we compared FOXA2-bound genes in NEPC and SCLC. We identified an overlap with more than 3,500 genes shared between the 2 neuroendocrine tumors that regulate pathways involved in neurogenesis and differentiation (Figure 2F). These data suggest that FOXA2 prefers to bind to the same genomic loci regulating neuroendocrine-associated pathways irrespective of neuroendocrine tumor type. Integrating FOXA2 cistrome with transcriptomics in NEPC and CRPC cell lines, we verified that upregulated FOXA2-bound genes in NCI-H660 compared with 16D^CRPC^ were highly associated with neuronal pathways as expected (Supplemental Figure 2, G and H). To identify TFs that cooperate with FOXA2, we performed motif enrichment analysis in SCLC and NEPC and ranked the motifs based on the *P* value. We identified ASCL1 motifs to be highly enriched at FOXA2 binding sites (Figure 2G). Overlaying the FOXA2 cistrome on chromatin accessibility using an assay for transposase-accessible chromatin using sequencing (ATAC-Seq), we identified that FOXA2 binds to both open and closed chromatin regions (Supplemental Figure 2I), supporting its pioneering function. The majority of FOXA2 binding sites were found at enhancer regions (intron and intergenic) irrespective of the chromatin accessibility status (Supplemental Figure 2J). Performing motif enrichment analysis, we found that FOXA2 was preferentially bound near the ASCL1 motif in NEPC-accessible chromatin (Figure 2H). These data indicated that NEPC and SCLC share the same FOXA2 target genes that are potentially flanked by ASCL1.

### ASCL1 and FOXA2 cooperate to establish the neuronal identity.

**Figure 3:**
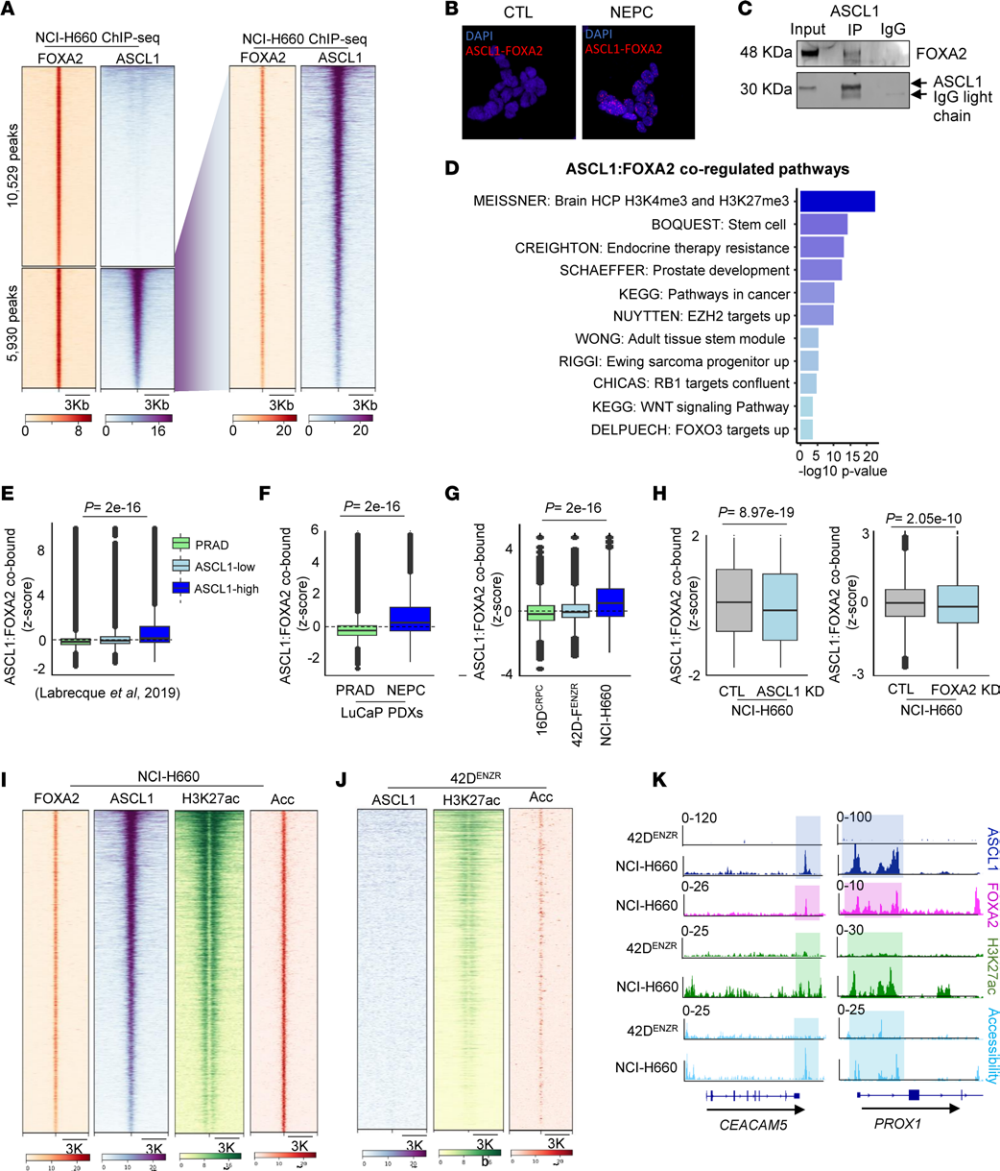
FOXA2 cooperates with ASCL1 in NEPC. (**A**) Heatmap of FOXA2 and ASCL1 ChIP-Seq binding intensity in NCI-H660; represents the regions co-occupied by FOXA2 alone or FOXA2 and ASCL1. (**B**) Proximity ligation assay (PLA) showing ASCL1 and FOXA2 interactions, visualized as red fluorescent dots, indicating molecular proximity (<40 nm), in NCI-H660. Nuclei are counterstained with DAPI (blue). Original magnification, ×20. (**C**) ASCL1 and FOXA2 interaction was measured by co-immunoprecipitation in NCI-H660. (**D**) Pathways regulated by ASCL1:FOXA2, shown as –log_10_ *P* value using gProfiler. (**E**) Box plot shows the expression of ASCL1:FOXA2 cobound genes in Adeno, ASCL1-low, and ASCL1-high NEPC patient datasets (3, 9). The middle solid line shows the median. Statistical significance was assessed using 2-tailed unpaired *t* test, followed by the Bonferroni method (ASCL1-high vs. PRAD *P* < 2 × 10^–16^). (**F**) Box plot shows expression of ASCL1:FOXA2 cobound genes in LuCaP PRAD and NEPC PDXs. The middle solid line shows the median. Statistical significance was assessed using a 2-tailed unpaired *t* test. (**G**) Box plot shows expression of ASCL1:FOXA2 cobound genes in 16D^CRPC^, 42D^ENZR^, 42F^ENZR^, and NCI-H660. The middle solid line shows the median. Statistical significance was assessed using a 2-tailed unpaired *t* test, followed by the Bonferroni method. (**H**) Box plot shows the expression of ASCL1:FOXA2 cobound genes in NCI-H660 following KD of ASCL1 (left) or FOXA2 (right). The middle solid line shows the median. Statistical significance was assessed using a 2-tailed unpaired *t* test. (**I**) Heatmap of FOXA2, ASCL1, H3K27ac ChIP-Seq, and chromatin accessibility (ATAC-Seq) shows binding and accessibility at ASCL1:FOXA2 cobound regions in NCI-H660. (**J**) Heatmap shows ASCL1 and H3K27ac binding intensity and chromatin accessibility in 42D^ENZR^ at ASCL1:FOXA2 cobound regions in NCI-H660. (**K**) IGV tracks represent ASCL1:FOXA2 target genes bound by ASCL1 and FOXA2 exclusively in NCI-H660 within the accessible region with H3K27ac mark.

Next, we explored the association between ASCL1 and FOXA2 by overlaying ASCL1 and FOXA2 ChIP-Seq. We observed up to 40% of FOXA2 binding sites were co-occupied by ASCL1 (Figure 3A) and identified 5,930 peaks annotated to 3,848 unique genes cobound by ASCL1:FOXA2 in NEPC (Figure 3A) and 4,329 peaks annotated to 3,166 unique genes in SCLC (Supplemental Figure 3A). A large portion (89% in NEPC and 92% in SCLC) of the cobound regions were annotated to enhancers (intron and intergenic) (Supplemental Figure 3, B and C). Motif analysis of the ASCL1:FOXA2 cobound region verified that FOXA2 and ASCL1 were the most enriched motifs in both NEPC and SCLC (Supplemental Figure 3D). We observed that ASCL1 and FOXA2 were in proximity to each other using proximity ligation assay (PLA) (Figure 3B and Supplemental Figure 3E). The ASCL1 and FOXA2 interaction was verified by immunoprecipitation (Figure 3C). Exploring the function of the ASCL1:FOXA2 cobound genes using gene set enrichment analysis (GSEA), we identified that ASCL1:FOXA2 genes were involved in pathways regulating endocrine therapy resistance, epigenetic, and stemness (Figure 3D). We identified 1,455 common genes cobound by ASCL1:FOXA2 in SCLC and NEPC. Similar pathways were enriched in cobound common genes, regulating stem cell and neuronal development (Supplemental Figure 3F). These data suggest that ASCL1 and FOXA2 cooperate in regulating the small cell tumor’s transcriptome and neuronal lineage specification.

Attesting to the human relevance, genes with cobound ASCL1:FOXA2 were upregulated in ASCL1-high patient NEPC tumors compared with ASCL1-low NEPC and PRAD (3) (Figure 3E), in NEPC LuCaP compared with PRAD (3) (Figure 3F), and in NCI-H660 compared with CRPC cell lines (Figure 3G). To determine the relative contribution of ASCL1 and FOXA2 at cobound genes, ASCL1 or FOXA2 was knocked down. The disruption of either ASCL1 or FOXA2 yielded the downregulation of ASCL1:FOXA2 cobound genes (Figure 3H and Supplemental Figure 3G), further validating the importance of ASCL1 and FOXA2 in regulating genes involved in lineage determination.

To understand if ASCL1:FOXA2 cobound regions are specific to terminal NEPC, we overlaid ATAC-Seq and H3K27ac on ASCL1:FOXA2 cobound regions. We found that ASCL1:FOXA2 regions were located within the accessible region flanked by H3K27ac marks (Figure 3I), suggesting that ASCL1:FOXA2 is specific to terminal NEPC. In agreement, we observed a lack of ASCL1 binding in these regions in neuroendocrine-like cell line 42D^ENZR^, located in closed chromatin lacking H3K27ac marks (Figure 3, J and K), in alignment with their expression observed in NEPC when compared with neuroendocrine-like and CRPC cell lines (Figure 3G). Of note, ASCL1 binding sites were deserted from FOXA2 in neuroendocrine-like cells (Supplemental Figure 3F). These results indicate that ASCL1 cooperates with FOXA2 to regulate terminal neuroendocrine genes in NEPC.

### ASCL1 and FOXA2 coregulate TF PROX1.

**Figure 4:**
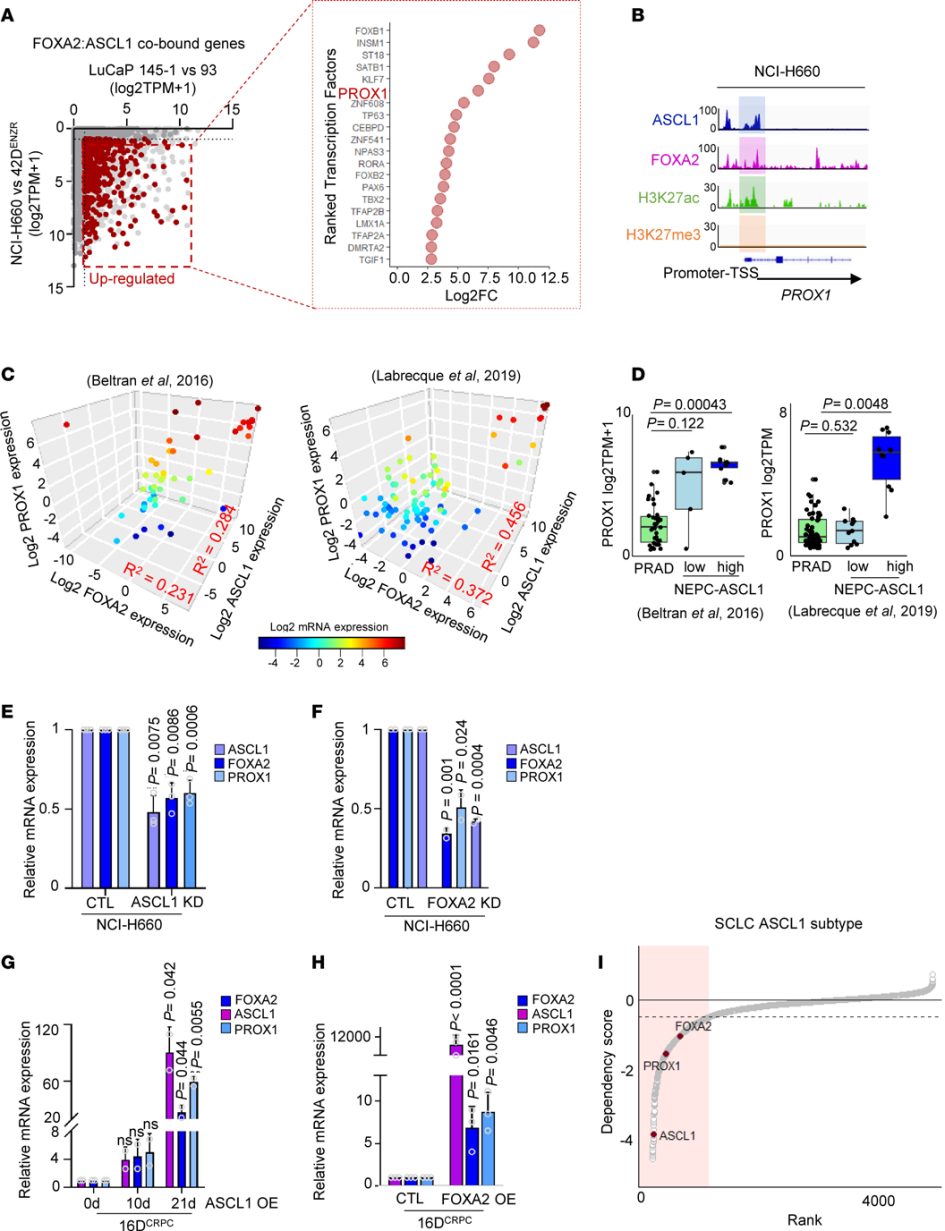
ASCL1 and FOXA2 coregulate PROX1. (**A**) Dot plot shows the fold-change in transcript abundance of FOXA2:ASCL1 cobound genes, *x* axis comparing NCI-H660 (ASCL1^+^FOXA2^+^) versus 42D^ENZR^ (ASCL1^+^FOXA^–^) cell lines and *y* axis comparing LuCaP 145-1 (ASCL1^+^FOXA2^+^) versus LuCaP 93 (ASCL1^+^FOXA2^–^) PDXs (left panel). Top upregulated TFs were ranked based on the highest log_2_ fold-change expression (right panel). (**B**) IGV tracks show ASCL1, FOXA2, H3K27ac, and H3K27me3 binding at the PROX1 promoter in NCI-H660. (**C**) The correlation between PROX1 expression with FOXA2 and ASCL1 in patient datasets (3, 9), with each dot representing a patient tumor and the color reflecting PROX1 mRNA expression; *P* < 0.05, with significance assessed by 2-tailed unpaired *t* test. (**D**) PROX1 expression in patient datasets (3, 9), with significance assessed using 2-tailed unpaired *t* test. Bonferroni’s correction was applied to adjust for multiple comparisons. (**E**) Relative mRNA expression of ASCL1, FOXA2, and PROX1 in NCI-H660 (CTL) following ASCL1 KD. Data are reported relative to CTL, mean ± SD; with significance assessed by 2-tailed unpaired *t* test. (**F**) Relative mRNA expression of ASCL1, FOXA2, and PROX1 in NCI-H660 (CTL) following FOXA2 KD. Data are reported as described in **E**. (**G**) Relative mRNA expression of ASCL1, FOXA2, and PROX1 normalized to GAPDH in 16D^CRPC^ (CTL) and following OE of ASCL1 at 10 and 21 days using a Dox-inducible system. Data are reported relative to day 0, and significance is assessed using a 2-tailed unpaired *t* test. (**H**) Relative mRNA expression of ASCL1, FOXA2, and PROX1 in 16D^CRPC^ (CTL) and following OE of FOXA2, with significance assessed by 2-tailed unpaired *t* test. (**I**) Identification of significant dependencies in the SCLC ASCL1 subtype using a loss-of-function CRISPR/Cas9 screen. Genes within the shaded red region are identified as significant dependencies with a dependency score of < –0.5.

To identify ASCL1:FOXA2 coregulated TFs, we compared the expression of ASCL1:FOXA2 cobound genes in NEPC cell lines and PDXs, in which both ASCL1 and FOXA2 (ASCL1^+^FOXA2^+^) are expressed, versus models where ASCL1 is expressed alone (ASCL1^+^FOXA2^–^). We focused on genes commonly upregulated in the ASCL1^+^FOXA2^+^ models compared with ASCL1^+^FOXA2^–^. Our data revealed a number of TFs upregulated in the ASCL1^+^FOXA2^+^, many of which were previously associated with important processes in SCLC, such as PROX1 (40) (Figure 4A). ASCL1 and FOXA2 bound to *PROX1* promoter that was highly acetylated at H3K27 and lacking H3K27me3 (Figure 4B), which was not observed in neuroendocrine-like 42D^ENZR^ (Supplemental Figure 4A). We also observed high H3K27me3 at *PROX1* promoter in 42D^EZNR^ and LuCaP 93 (Supplemental Figure 4A). This pattern of ASCL1 binding and histone marks correlated with PROX1 expression across these models, where PROX1 expression was observed when both ASCL1 and FOXA2 were expressed (Figure 4A).

The expression of ASCL1, FOXA2, and PROX1 positively correlated in prostate cancer patient datasets (3, 9, 46) (Figure 4C) as well as lung cancer (47) (Supplemental Figure 4B). PROX1 expression was elevated in NEPC compared with PRAD (Supplemental Figure 4C) and in SCLC compared with NSCLC (47) (Supplemental Figure 4D). This increase was exclusive to the ASCL1-high NEPC population (Figure 4D and Supplemental Figure 4E). Contrary to previous work (48), we did not observe any PROX1 induction by ARPI in cell lines (Supplemental Figure 4, F and G), or patients (41) (Supplemental Figure 4H), nor did we detect AR binding toPROX1 promoter (Supplemental Figure 4I). To determine whether ASCL1 and FOXA2 are required for PROX1 expression, we examined PROX1 expression following loss of either ASCL1 or FOXA2 in NEPC. We observed that loss of ASCL1 or FOXA2 led to decreased PROX1 expression (Figure 4, E and F) while their overexpression led to its increase (Figure 4, G and H, and Supplemental Figure 4J). Together, these data suggest that ASCL1 and FOXA2 work in concert to reinforce neuron-specific programs and lineage maturation by inducing the expression of lineage-specific TFs including PROX1. Interestingly, in the SCLC ASCL1-driven subtype, loss-of-function CRISPR/Cas9 screen (49) identified PROX1 and FOXA2 as important dependencies (Figure 4I). These findings highlight the critical role of PROX1 and FOXA2 in the ASCL1-driven subtype, further underscoring their importance in the regulation of neuronal differentiation programs.

### PROX1 is required for the neuronal lineage identity and tumor growth.

**Figure 5:**
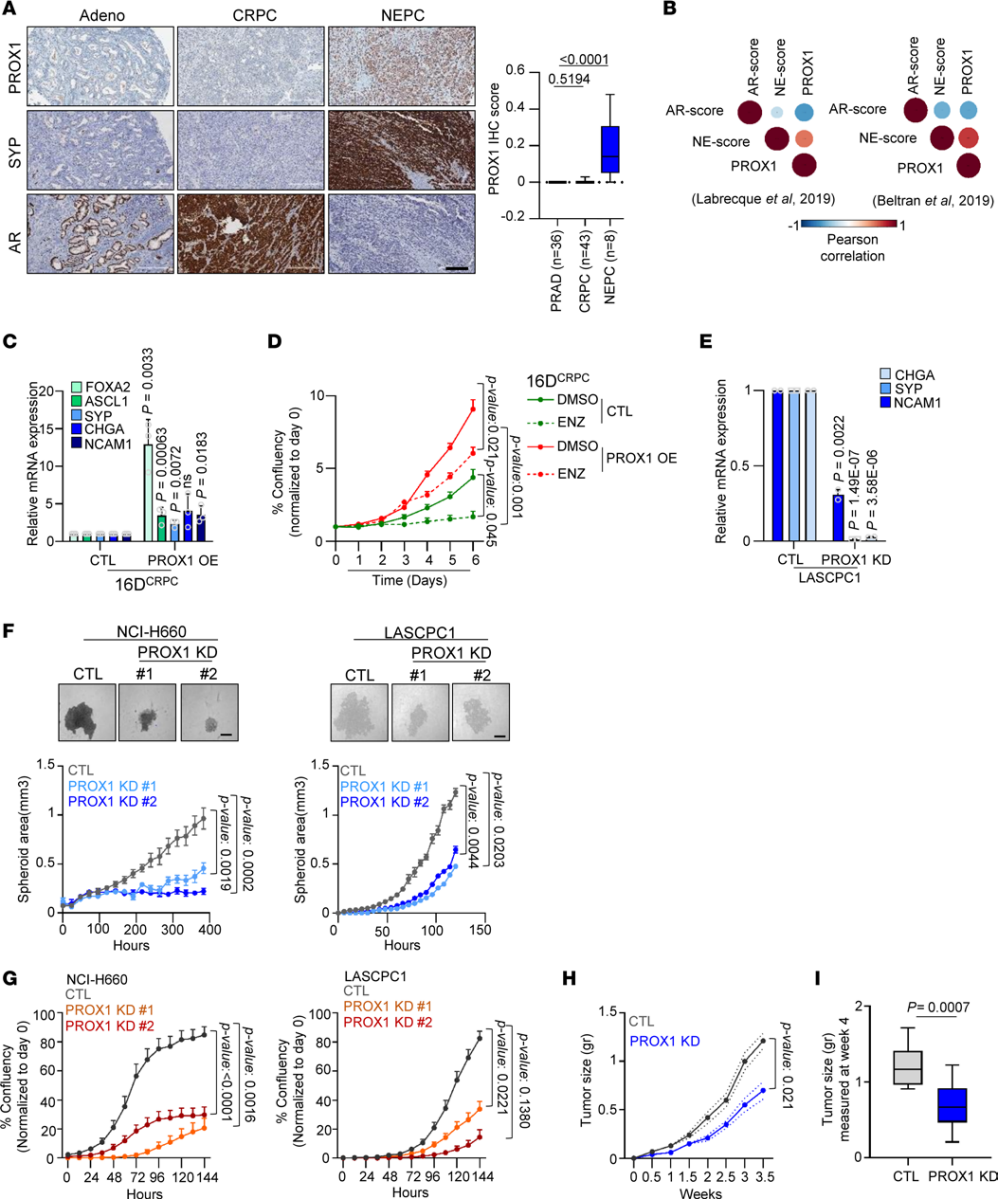
PROX1 is required for neuroendocrine phenotype characteristics and proliferation. (**A**) IHC staining for Adeno, CRPC, and NEPC clinical samples. Scale bar: 200 μm (left panel). PROX1 staining intensity was quantified; mean ± SD with significance was measured using 1-way ANOVA statistical test followed by Dunnett’s test (right panel). (**B**) Pearson’s correlation between PROX1 mRNA expression and AR or neuroendocrine (NE) score in patient datasets (3, 9). (**C**) Relative mRNA expression normalized to GAPDH in 16D^CRPC^ (CTL) and following PROX1 OE, reported relative to CTL (mean ± SD). Two-tailed unpaired *t* test. (**D**) Proliferation of 16D^CRPC^ (CTL) and PROX1 OE treated with DMSO or ENZ for 6 days, reported normalized to day 0, with significance calculated at day 6 using unpaired 2-tailed *t* test. (**E**) Relative mRNA expression of NE genes in LASCPC1 (CTL) and PROX1 KD. Data are reported relative to CTL (mean ± SD; *n* = 3). Two-tailed unpaired *t* test. (**F**) Images of second-generation spheroids at day 5 of NCI-H660 (left side) and LASCPC1 (right side) CTL and PROX1 KD. Scale bar: 100 μm (upper panel). Quantification of spheroid data reported as mean ± SD. *P* value calculated using 1-way ANOVA followed by Dunnett’s test (lower panel). (**G**) Proliferation of NCI-H660 (left side) and LASCPC1 (right side) CTL and PROX1 KD. Confluence is shown as a percentage, normalized to day 0. *P* values were calculated using 1-way ANOVA statistical test followed by Dunnett’s test. (**H**) Tumor size in LASCPC1 (CTL) and PROX1 KD reported as mean gram ± SEM, with significance evaluated at the endpoint (week 4). *n* = 10 animals per arm; 2-tailed unpaired *t* test. (**I**) Tumor size reported at the endpoint (week 4) in LASCPC1 (CTL) and PROX1 KD (*n* = 10). Significance evaluated using a 2-tailed paired *t* test.

PROX1 expression was verified in NEPC specimens using IHC along with neuroendocrine marker SYP (Figure 5A). PROX1 expression positively correlated with neuroendocrine score and negatively correlated with AR score in prostate cancer patient data sets (3, 9, 46) (Figure 5B). In alignment, PROX1 was expressed in NEPC cell lines along with neuroendocrine genes such as *NCAM1* and *BRN2* (Supplemental Figure 5A). Overexpressed PROX1 in CRPC cell line 16D^CRPC^ (Supplemental Figure 5B) was sufficient to increase neuroendocrine markers’ expression (Figure 5C) and induced resistance to ENZ treatment (Figure 5D). Silencing PROX1 in NEPC models using shRNA led to downregulation of neuroendocrine gene expression (Figure 5E), which was validated with Western blot (Supplemental Figure 5C). Furthermore, GSEA revealed that loss of PROX1 suppressed pathways specific to neurogenesis and neuronal development (Supplemental Figure 5D), reduced spheroid formation as a measure of functional properties of stemness (Figure 5F), and decreased cell proliferation capacity in vitro (Figure 5G). The effect of loss of PROX1 on cell proliferation was translated in vivo, where xenografts bearing PROX1 knockdown grew at a slower rate (Figure 5H and Supplemental Table 1) and were smaller (Figure 5I) compared with the control group. These data suggest that PROX1 can induce neuronal lineage identity and proliferation.

## Discussion

TFs recognize and bind to specific motifs on the DNA, which consist of a few base pairs. Although these motifs are spread across the genome, only certain ones are bound by TFs in specific cell types or lineages. ASCL1 is known for its critical role in neurogenesis, particularly in making cell fate decisions. We characterized ASCL1 binding sites in a set of terminally differentiated NEPC and in treatment-induced tNEPC models. We observed that ASCL1 binding patterns differ at these stages, with ASCL1 binding to distinct promoters in terminal NEPC compared with the metaplastic ARPI-resistant stage. The decision-making process of ASCL1 regarding its targets is complex, as TFs do not bind to all their potential motifs throughout different stages. The variability in ASCL1 expression and its target expression across different cell lines and PDXs suggests that these models are at different stages of the disease trajectory. This may indicate that the level of ASCL1 expression may result in ASCL1 playing either a pro-proliferative or pro-differentiation role, depending on the cellular context and availability of cofactors. These observations highlight the importance of identifying the unique targets of ASCL1 in terminal NEPC and its cofactors facilitating its target genes. We found that ASCL1 regulates several well-known pro-neuronal factors, including FOXA2. It is important to note that the expression of ASCL1 is induced by ARPIs (16). However, this is not the case for FOXA2. ASCL1 expression continues to rise, potentially aligning with the timing of relapse when FOXA2 expression becomes apparent.

Previous studies have suggested that FOXA2 acts as both a regulator and a marker of neuroendocrine differentiation (17, 34, 39, 50) and also acts as a pan-plasticity factor, facilitating the development of various AR-independent CRPC lineages (51). Despite these findings, the detailed molecular functions of FOXA2 and the identities of its associated TFs remain unclear. Our analysis provided insights into FOXA2 binding pattern and transcriptional regulation, demonstrating that FOXA2 targets are conserved between SCLC and NEPC. Our data suggest that when FOXA2 is expressed, it preferentially targets the same genomic regions, regardless of the cell of origin, and reinforces neuroendocrine characteristics. Importantly, we identified the ASCL1 motif in close proximity to the FOXA2 binding site, providing evidence of their collaborative interaction. Our data suggest that FOXA2 and ASCL1 interact closely to coordinate transcriptional programs essential for maintaining the terminal neuroendocrine phenotype. Our data highlighted a unique dynamic between ASCL1 and FOXA2, where ASCL1 regulates FOXA2 expression in a subset of NEPC. Following the increased expression of FOXA2, ASCL1 coordinates with FOXA2 in regulating neuron-specific genes. ASCL1:FOXA2 cobound regions were open and active when both TFs were expressed, in contrast to where ASCL1 was expressed alone and acting as a cofactor. This interaction could be important for monitoring disease progression and therapeutic outcomes.

Mechanistically, we showed that ASCL1 and FOXA2, through direct transcriptional regulation of PROX1, mediate neuronal lineage fate specification. During embryonic development, PROX1 supports survival, final specification, and cell fate determination (52, 53). Here, we showed that PROX1 induces neuroendocrine phenotype. We found that the loss of PROX1 abrogated neuronal stem cell identity. Additionally, we demonstrated that PROX1 helps maintain neuronal fate and supports tumor growth.

## Methods

*Sex as a biological variable*. In this study, only male mice (NSG NOD.Cg-Prkdcscid Il2rgtm1Wjl/SzJ, aged 6–8 weeks) were used. Prostate cancer is a male-specific disease, and thus, male mice were selected to accurately model the biological and clinical characteristics of the disease. The use of male mice ensures relevance to human prostate cancer, and findings are not expected to vary significantly across sexes, as prostate cancer primarily affects males. This choice aligns with the biological specificity of the disease.

*Cell lines and tissue culture*. NCI-H660 (catalog CRL-5813), and LASCPC1 (catalog CRL-3356) cell lines were obtained from ATCC. HEK293T (catalog R70007) was obtained from Thermo Fisher Scientific. CRPC (16D^CRPC^) and ENZ-resistant AR^+^ neuroendocrine-like (42D^ENZR^ and 42F^ENZR^) cell lines were generated from LNCaP cells, previously detailed by our group (16, 27, 54, 55). NCI-H660 and LASCPC1 cell lines were cultured in HITES medium following ATCC recommendation. LNCaP-driven and ENZ-resistant cell lines were cultured in RPMI 1640 medium (Thermo Fisher Scientific, catalog 11875093) plus 5% FBS (Gibco, catalog A3160701). HEK293T cells were cultured in DMEM (Thermo Fisher Scientific, catalog 11995-065) supplemented with 5% FBS and 2 mM l-glutamine. Cells were treated with DMSO or 10 μmol/L of ENZ. All cell lines were cultured in humidified atmospheric air with 5% CO_2_ at 37°C. When indicated, cells were treated with 10 μmol/L of ENZ. Cultures were assessed for mycoplasma weekly, and all cell lines were authenticated by short tandem repeat profiling.

*Animal study*. The animal study was conducted adhering to the Animal Care Committee at The University of British Columbia (no: A20-0232). Male 6- to 8-week-old mice (NSG NOD.Cg-Prkdcscid Il2rgtm1Wjl/SzJ; Jackson Laboratory) were maintained in ventilated cages (4 mice per cage), with constant humidity of 25%–47% and temperature of 21°C–22°C under 12-hour light/12-hour dark cycle with ad libitum access to rodent chow diet and drinking water. Prostate cancer is a male-specific disease, and thus only male mice were used to accurately model the biological and clinical characteristics of the disease.

Mice were inoculated with an established LASCPC1 shControl or LASCPC1 shPROX1 cell line (1 × 10^6^ cells per 1 site, right flank), 10 mice per arm. Tumor growth was monitored and measured twice per week in a blinded fashion. The mice were sacrificed at the end of the study (tumor reached 10% of body weight, a body weight loss of more than 15%, or tumor volume of 2,000 mm^3^). Tumor volume was calculated with the formula: V = (π[length **×** width **×** height])/6. At the experimental endpoint, mice were euthanized using isoflurane inhalant anesthesia followed by CO_2_ euthanasia as described in The University of British Columbia SOP ACC 03-2012.

*Plasmids*. The plasmids used to knock down PROX1 were purchased from MilliporeSigma (Sigma MISSION Predesigned shRNA clone) with the following sequence: PROX1 shRNA #1 targeted sequence 5′-TTTCCAGGAGCAACCATAATT (TRCN0000232122), PROX1 shRNA #2 targeted sequence 5′-GGCTCTCCTTGTCGCTCATAA (TRCN0000232124), PROX1 shRNA #3 targeted sequence 5′- GAAGTTGCTCAGATCACATTA (TRCN0000232125).

The plasmids used to knock down FOXA2 were purchased from MilliporeSigma (Sigma MISSION Predesigned shRNA clone) with the following sequence: FOXA2 shRNA #1 targeted sequence 5′-GCAAGGGAGAAGAAATCCATA (TRCN0000014913), FOXA2 shRNA #2 targeted sequence 5′-GAACGGCATGAACACGTACAT (TRCN0000014915), FOXA2 shRNA #3 targeted sequence 5′-CTACGCCAACATGAACTCCAT (TRCN0000014916).

ASCL1 Tet-on inducible system vectors were pTet-O-ASCL1-T2A-PuroR (Addgene, catalog 162345) and FUW-M2rtTA (Addgene, catalog 20342).

MISSION pLKO.1-puro nonmammalian shRNA control plasmid DNA (MilliporeSigma catalog SHC002) was used to generate shControl cell lines. Lentiviral packaging plasmids pMD2 (Addgene, catalog 8454) and psPAX7 (Addgene, catalog 12260) were purchased from Addgene. All plasmids were analyzed for correct insertion by Sanger sequencing before use.

*Lentiviral vector preparation and infection*. HEK293T cells were plated 1 day before the transfection at 80% confluence in a complete medium. Cells were transfected with the PROX1, FOXA2, Tet-on ASCL1, Tet-on CTL plasmids, pMD2.G and psPAX7, at a ratio of 4:1:2. Medium was changed 24 hours following transfection to complete medium. The virus was collected at 48 and 72 hours after transfection using a 0.45 μM Sterifilip.

For transduction, 200,000 cells were plated in 12-well plates in 500 μL appropriate standard serum-free media for the cell type, supplemented with 8 μg/mL polybrene. A total of 500 μL of viral supernatant was added to each well. At 24 hours later, the medium was replaced with a complete medium, and 0.5 μg/mL puromycin was added for the selection.

For Tet-on ASCL1, equal volumes (1 mL) of pTet-O-ASCL1-T2A-PuroR and FUW-M2rtTA viruses were added to 16D^CRPC^ cell lines in suspension and centrifuged at 1,000*g* with 5 μg/mL Polybrene Infection Reagent (MilliporeSigma, catalog TR-1003-G) for 2 hours at room temperature. At 48 hours after infection, transduced cells underwent puromycin selection (2 μg/mL) for 7 days. ASCL1 overexpression was induced by 1 μM doxycycline (MilliporeSigma, catalog D5207-1G) treatment for 10 days and 28 days, respectively. Doxycycline was refreshed every 48 hours.

*Western blot*. Total proteins were extracted from cells and washed with 1× cold PBS followed by adding RIPA buffer (Thermo Fisher Scientific, catalog PI89901) supplemented with 1× concentration of cOmplete EDTA-free protease inhibitors cocktail (Roche, catalog 11836170001) and phosphatase inhibitors (PhosSTOP, Roche, catalog 4906845001). Protein concentrations were measured using BCA assay (Thermo Fisher Scientific, catalog 23225). Next, samples were boiled for 5 minutes in 1× SDS sample buffer and run on an SDS-PAGE gel. The gel was transferred to a PVDF membranes (MilliporeSigma, catalog IPVH00010) with a pore size of 0.45 μm, blocked with Odyssey Blocking Buffer (LI-COR, catalog 15590545; 1:2), and probed with primary antibodies at dilution indicated below. Membranes were imaged using the LI-COR Odyssey Imaging System with Li-COR Image Studio (version 4.2) software. The following parameters were used to scan the membranes: channels 700 for mouse and 800 for rabbit secondary antibodies; resolution of 169 μm; intensities of channel 700: 3.0 and channel 800: 1.5–3.0.

The following antibodies were used for immunoblotting: AR (Cell Signaling Technology, catalog 5153; clone D6F11; 1:1,000), FOXA2 (Cell Signaling Technology, catalog 8186S [D56D6] 1:1,000), PROX1 (R&D Systems, Bio-Techne, catalog AF2727), NCAM1 (Cell Signaling Technology, catalog 99746S), and BRN2 (Cell Signaling Technology, catalog 12137S), and β-actin (MilliporeSigma A2228; clone AC-74; 1:25,000) was used as a loading control. IRDye 800CW donkey anti-rabbit (LI-COR, catalog 926-32213; 1:10,000) and IRDye 680CW donkey anti-mouse (LI-COR, catalog 926-68072; 10,000) were used as secondary antibodies.

*Co-immunoprecipitation*. For immunoprecipitation, cells were washed with PBS and lysed in IP Lysis Buffer (Thermo Fisher Scientific, catalog 87787) supplemented with 1× cOmplete EDTA-free protease inhibitors cocktail and phosphatase inhibitor. After incubating on ice for 45 minutes, cell lysates were centrifuged at maximum speed for 25 minutes at 4°C to remove cell debris. Protein concentration was measured using BCA protein assay kit. A total of 1,000 μg of proteins was incubated on a rotating platform overnight at 4°C with 20 μL of Magna ChIP Protein A+G Magnetic Beads (MilliporeSigma, catalog 16-663) plus ASCL1 antibody (5 μg, BD Biosciences, catalog 556604). As an isotype control, 80 μg of proteins was incubated with A+G beads and 5 μg of normal mouse IgG (Santa Cruz Biotechnology, catalog sc-2025) in parallel with the sample. Another 80 μg of lysates was set aside and stored at –20°C in a freezer overnight as an input control. After overnight incubation of sample and isotype control, beads were washed 3 times with IP Lysis Buffer, and samples were eluted in SDS sample buffer.

For Western blot, input control, isotype control, and sample were boiled for 15 minutes in SDS sample buffer and run on a 10% SDS-PAGE gel. The transfer was performed using a 0.45 μm PVDF membrane. After transfer, the membrane was blocked with Odyssey Blocking Buffer (LI-COR, catalog 15590545; 1:2) and probed with ASCL1 (BD Biosciences, catalog 556604; 1:500) and FOXA2 (Cell Signaling Technology, catalog 8186S; 1:2,000) primary antibodies. The membrane was imaged using the LI-COR Odyssey Imaging System with LI-COR Image Studio (version 4.2) software. The following parameters were used to scan the membrane: channel 700 for mouse and channel 800 for rabbit secondary antibodies; resolution of 169 μm; channel intensities: 3.0.

*PLA and quantification*. NCI-H660 cells were cultured in RPMI 1640 medium supplemented with 5% FBS, 1× Insulin-Transferrin-Selenium (Gibco, catalog 41400-045), 10 nM hydrocortisone, 10 nM β-estradiol, and 4 mM l-glutamine (Gibco, catalog 35050-061) in humidified atmospheric air with 5% CO_2_ at 37°C.

Plating for PLA was performed using an 8-well Chamber slide system (Thermo Fisher Scientific, catalog 154453PK). Wells were coated with poly-l-lysine (MilliporeSigma, catalog 25988-63-0) for 5 hours at 37°C before plating 40,000 NCI-H660 cells per well and incubating at 37°C overnight. The attached cells were fixed with 4% paraformaldehyde in 1× PBS (Thermo Fisher Scientific, catalog AAJ19943K2) at room temperature for 20 minutes with gentle shaking and permeabilized with 0.5% Triton X-100 in PBS for 15 minutes. After blocking with 3% BSA and 0.1% Triton X-100 in PBS at room temperature for 30 minutes, cells were incubated with FOXA2 (Cell Signaling Technology, catalog 8186S) and ASCL1 (BD Biosciences, catalog 556604) antibodies, diluted 1:100 in 2.5% BSA in PBS, at 4°C overnight. Cells were washed 3 times with PBS between steps. On the next day, PLA was performed using the Duolink In Situ Detection Reagents Red kit (MilliporeSigma, catalog DUO92008-30RXN) with the corresponding anti-rabbit PLUS (MilliporeSigma, catalog DUO92002-30RXN) and anti-mouse MINUS (MilliporeSigma, catalog DUO92004-30RXN) PLA probes following manufacturer’s protocol.

PLA fluorescence was detected on an Olympus FV3000 scanning confocal microscope system using the 405 nm and 561 nm channels. The Olympus cellSens software was used to combine *Z*-stacks and normalize image intensities for downstream analysis. DAPI (405 nm) and PLA (561 nm) channels were split using the ImageJ software (NIH) and the Fiji image processing package. The CellProfiler software was used to calculate puncta per nucleus. R was used to perform the final cleanup and generate quantification plots using dplyr, ggplot2, and ggpubr packages.

*Quantitative real-time PCR*. RNA was extracted from cells using the RNAzol RT Reagent (Molecular Research Center, Inc., catalog RN190) following the manufacturer’s protocol. Reverse transcription was performed using 1 μg RNA and LunaScript RT SuperMix (New England Biolabs, catalog M3010L) containing random hexamers, oligo-dT primers, dNTPs, RNase inhibitor, and reverse transcriptase. Synthesized cDNA (30 ng/μL) was amplified for quantitative real-time PCR on a QuantStudio 7 Pro Real-Time PCR System using the Luna Universal qPCR Master Mix (New England Biolabs, catalog M3003L) with gene-specific primers. GAPDH was used for normalization. Fold-changes in mRNA expression levels were calculated using the comparative Ct method (ΔΔCt). Real-time PCR was performed using an ABI ViiA7. Primer sequences used are as follows: ASCL1: Fwd: 5′-GAGCAGGAGCTTCTCGACTTC, Rev: 5′-GATGCAGGTTGTGCGATCAC; BRN2: Fwd: 5′-ACACTGACCGATCTCCACGCAGTA, Rev: 5′-GAGGGTGTGGGACCCTAAATATGA; NSE: Fwd: 5′-GAACTATCCTGTGGTCTCC, Rev: 5′-CGACATTGGCTGTGAACTTG; CHGA: Fwd: 5′-TCCAAGGCGCCAAGG, Rev: 5′-CATCTTCAAAACCGCTGTGTTTC; NCAM1: Fwd: 5′-GATGCGACCATCCACCTCAA, Rev: 5′-TCTCCGGAGGCTTCACAGGTA; OCT4: Fwd: 5′-GTCCGAGTGTGGTTCTGTA, Rev: 5′-CTCAGTTTGAATGCATGGGA; SOX2: Fwd: 5′-TGCGAGCGCTGCACA, Rev: 5′-TCATGAGCGTCTTGGTTTTCC; FOXA2: Fwd: 5′-GGAGCAGCTACTATGCAGAGC, Rev: 5′-CGTGTTCATGCCGTTCATCC; PROX1: Fwd: 5′-AAAGGACGGTAGGGACAGCAT, Rev: 5′-CCTTGGGGATTCATGGCACTAA.

*Spheroid assay*. Single-cell suspensions (1,000 cells/mL) were seeded onto ultralow-attachment plates and maintained in serum-free NeuroCalt NS-A Basal Medium (Human) (STEMCELL Technologies, catalog 05750), with the addition of 2% B27 (Thermo Fisher Scientific, catalog A3582801), 1% N2 (Thermo Fisher Scientific, catalog 17502048), 20 ng/mL basic fibroblast growth factor (STEMCELL Technologies, catalog 78003.1), 20 ng/mL epidermal growth factor (STEMCELL Technologies, catalog 78006.1), and 2 μg/mL heparin (STEMCELL Technologies, catalog 07980). The cells were cultured for 7 to 10 days to generate the first generation and for 7 days for the second generation. For serial passage, tumor spheres were collected using 40 μm cell strainers (Sarstedt catalog 83.3945.040), then dissociated into single cells using Accutase (STEMCELL Technologies, catalog 07922) for 10 minutes at room temperature. The tumor spheres were imaged with the IncuCyte S3.

*Proliferation*. Cells were first dissociated into single cells using Accutase (STEMCELL Technologies, catalog 07922) for 10 minutes at room temperature. Next, 2,000 cells/well were seeded in 96-well plates, allowed to attach overnight, treated with drug (when indicated), and imaged using the IncuCyte S3. Cell confluence was assessed using the IncuCyte Basic Analyzer with a minimum size filter of 400 μm^2^ for adherent cells and 100 μm^2^ for suspended cells. A minimum of 4 technical and 3 biological replicates were performed for each cell line/treatment.

*RNA-Seq and data analysis*. Total RNA was isolated from cell lines using the RNeasy Mini Kit (QIAGEN, catalog 74104). Library constructions were performed using the NEBnext Ultra ii Stranded RNA Library Prep Kit (New England Biolabs), and sequencing was performed on an Illumina NextSeq 500 (42 × 42 bp paired-end reads).

Data were demultiplexed using bcl2fastq2 Conversion Software (version 2.20), and the resultant read sequences were aligned to the hg38 human reference genome using STAR aligner (56). Differential expression was estimated using the DEseq2 package in Rstudio. For patient tumors, sequencing data were aligned to hg38 using TopHat, and the number of reads per gene was measured with HTSeq count. Gene counts (raw count data) were normalized and log-transformed using DESeq (57). Read counts per gene were determined using featureCounts (58). For visualization purposes, the data were *Z*-transformed per gene.

*ChIP-Seq*. Cell lines were cultured in their complete media as stated above. For FOXA2 ChIP-Seq, 10 million cells were cross-linked with 1% formaldehyde for 7 minutes at room temperature with shaking. Cross-linking reactions were quenched with 0.125 M glycine for 5 minutes and washed twice with ice-cold PBS. Following cell and nucleus lysis, nucleus pellets were resuspended in 500 μL of 1% SDS (50 mM Tris-HCl pH 8, 10 mM EDTA) and sonicated for 7 minutes using a Covaris E220 instrument (setting: 140 peak incident power, 5% duty factor, and 200 cycles per burst) in 1 mL adaptive focused acoustics fiber millitubes. Chromatin was immunoprecipitated with 10 μg of FOXA2 (R&D Systems, Bio-Techne, catalog AF2400) antibody. A total of 80 μg of chromatin was used for chromatin immunoprecipitation. ChIP-Seq libraries (100 ng DNA per sample) were constructed using the KAPA HyperPrep Kit with Illumina TruSeq indexes (Roche, catalog KK8510). Libraries were assessed for quality using gel electrophoresis, and libraries passing quality control (e.g., no primer dimers) were quantified using the KAPA Library Quantification Kit for Illumina (Roche, catalog KK4824). Libraries were sequenced using the Illumina NextSeq 500 (75 bp single-end reads).

*ChIP-Seq and ATAC-Seq data analysis*. FastQC version 0.11.9 (59) was used for quality control analysis of FASTQ files, and in the case of ATAC-Seq FASTQ, adapter sequences were removed using Cutadapt v1.18. Raw reads were aligned to the hg38 reference (human) genome using BWA-MEM software (version 0.7.17) (60) with default parameters. Alignments with mapping quality less than 30 were filtered out (leaving only uniquely mapped reads). The SAM files were converted to BAM files using Samtools software (version 1.1.2) (61). Peaks were called using MACS2 (version 2.2.7.1) (62) with FDR *q* value = 0.05 using the narrow peak caller for accessible DNA, ASCL1, and FOXA2, while the broad peak calling was used for H3K27ac. Deeptools v2.30.0 (63) program suite was used for the visualization of data. Bedtools v2.28.0 (64) program suite was used to generate shared and unique peaks between various ChIP-Seq and ATAC-Seq samples.

For measuring chromatin accessibility, ATAC peaks were called from reads with a template length between 40 and 120 bp. Annotation was followed using HOMER (64). Bedtools computed the intersections between peak files, where 2 peaks were considered overlapping if the length of the overlapping region was more than 50% of the length of either peak. bigWig files were generated using deepTools software and were used to visualize heatmaps. Using Deeptools, heatmaps were next generated using *computeMatrix* and *plotHeatmap* programs.

*Motif analysis*. MACS files were generated using BED files after peak calling. They were converted to FASTA files with ±50 bp window around the center of each peak and Bedtools program suite for both ChIP-Seq and ATAC-Seq peaks. The motif enrichment analysis was performed using FASTA file as input and findMotifs.pl HOMER program suite v4.10 (65). To identify significantly enriched motifs under a given condition, motifs were ranked by log *P* value, and the difference in rank was plotted.

*Gene ontology and pathway analysis*. Pathway analysis using GSEA software from the Broad Institute (Massachusetts Institute of Technology) was used to identify functions of differentially expressed genes within the Molecular Signatures Database (version 7.1) (66, 67). The tool was run in classic mode to identify significantly enriched biology pathways. Pathways enriched with a nominal *P* < 0.05 and FDR < 0.25 were considered significant. Single-sample GSEA was carried out using gProfiler (68).

*Statistics*. All statistical analysis and visualization was performed using GraphPad Prism (version 8), unless otherwise specified. FDR and *P* values for all GSEA were carried out by GSEA software (version 7.1) or gProfiler web server (68) and for motif analysis by HOMER using hypergeometric test. Representative data shown in micrographs such as Western blot have been repeated 3 times with independent biological samples unless otherwise indicated. Data acquired from the IncuCyte S3 for proliferation analysis have been repeated with 3 independent biological samples. Representative data shown for all in vitro experiments were repeated at least 2 times unless otherwise indicated. In bar graphs, box-and-whisker, and violin plots, unpaired, 2-tailed *t* tests were performed to analyze statistical significance between groups using GraphPad Prism (version 8), and Bonferroni’s correction was applied to adjust for multiple comparisons. For longitudinal profiling experiments, a 2-tailed *t* test was performed to determine the statistical difference at the final time point using GraphPad Prism (version 8). For more than 2 groups, 1-way ANOVA test was performed followed by Dunnett’s test. Box plots show the interquartile range, median (line), and minimum and maximum (whiskers). *P* < 0.05 was considered significant. All exact *P* values are listed in the corresponding Supporting Data Values.

### Study approval

The animal experiments adhered to protocols approved by the Animal Care Committee at The University of British Columbia (approval no. A16-0246).

### Data availability

The RNA-Seq and ChIP-Seq data generated in this study have been deposited in the NCBI GEO database under the accessions GSE273956 and GSE273957. Publicly available LuCaP PDX RNA-Seq data used in this study are available in the GEO dataset under the accession code GSE126078 (3). Publicly available RNA-Seq data used in this study are available from the Beltran 2016 cohort from Beltran et al. (9, 69), the Labrecque 2019 from Labrecque et al. (3), the Abida 2019 from Abida et al. (46), as well as the SCLC cohort from Cancer Cell Line Encyclopedia portal (https://sites.broadinstitute.org/ccle/) (47). The publicly available ASCL1 ChIP-Seq in LuCaP PDXs used in this study is available in the GEO dataset under the accession code GSE156290 (18). The publicly available ASCL1 ChIP-Seq in NCI-H660 used in this study is available in the GEO dataset under the accession code GSE156292 (18). The ASCL1 ChIP-Seq in NCI-H889 used in this study is available in the GEO dataset under the accession code GSE69394 (20). FOXA2 ChIP-Seq in NCI-H889 used in this study is available in the GEO dataset under the accession code GSE151002 (40). C4-2B PRNB publicly available RNA-Seq used in this study is available in the GEO dataset under the accession code GSE225013. RNA-Seq of C4-2 treated with ENZ used in this study is available in the GEO dataset under the accession code GSE159548 (70). 16D^CRPC^, 42D^ENZR^, NCI-H660 control, and 16D^CRPC^ treated with ENZ and NCI-H660 siASCL1 RNA-Seq used in this study are available in the GEO dataset under the accession code GSE183199 (16), 42F^ENZR^ under the accession code GSE138460 (55), and 49C^ENZR^ and 49F^ENZR^ under the accession code GSE267309. 42D^ENZR^ and NCI-H660 ATAC-Seq used in this study are available in the GEO dataset under the accession code GSE183199 (16). 42D^ENZR^ H3K27me3 and H3K27ac ChIP-Seq used in this study are available in the GEO dataset under the accession code GSE138460 (55). 42D^ENZR^, H3K27me3, and H3K27ac ChIP-Seq in LuCaP PDXs used in this study are available in the GEO dataset under the accession code GSE161948 (42). SCLC ASCL1 subtype CRISPR/Cas9 loss-of-function screen was downloaded from He et al. (49). All data points shown in graphs and values behind reported means are provided in a separate Excel file of Supporting Data Values.

## Author contributions

SN, TN, dg, and AZ conceptualized and designed the study. SN and TN are co–first authors, with authorship order determined based on their respective contributions: TN performed the majority of the experiments and data acquisition, while SN analyzed the data and prepared and wrote the manuscript. Both contributed equally to the design of the study. TN generated cell lines, and performed experiments, acquired data, and analyzed data, SN, NT, and DG analyzed data and generated figures. DG performed peak calling, quality control, and visualization for ChIP-Seq and ATAC-Seq as well as processing of the public data. JMS processed the RNA-Seq. NT, CJC, and MK performed experiments, acquired data, and analyzed data. OS conducted the in vivo study. SN wrote the manuscript. SN, TN, NT, MK, DG, and AZ reviewed and edited the manuscript. All authors provided intellectual input and vetted and approved the final manuscript.

## Supplementary Material

Supplemental data

Unedited blot and gel images

Supplemental table 1

Supporting data values

## Version 1 10/29/2024

In-Press Preview

## Version 2 12/06/2024

Electronic publication
