# ASCL1 regulates and cooperates with FOXA2 to drive terminal neuroendocrine phenotype in prostate cancer

**DOI:** 10.1172/jci.insight.196861

**Published:** 2025-08-08

**Authors:** Shaghayegh Nouruzi, Takeshi Namekawa, Nakisa Tabrizian, Maxim Kobelev, Olena Sivak, Joshua M Scurll, Cassandra Jingjing Cui, Dwaipayan Ganguli, Amina Zoubeidi

Original citation: *JCI Insight*. 2024;9(23):e185952. https://doi.org/10.1172/jci.insight.185952

Citation for this corrigendum: *JCI Insight*. 2025;10(15):e196861. https://doi.org/10.1172/jci.insight.196861

The Methods have been updated to indicate the source of the tissue microarray data. The added text is below.

*Tissue microarray*. The tissue microarray was generated in 2016 by the pathology core at the Vancouver Prostate Centre (VPC), using British Columbian Canadian prostate cancer specimens obtained from surgeries performed at Vancouver General Hospital and UBC Hospital.

The HTML and PDF versions have been updated online.

## Version 1 08/08/2025

Electronic publication

