# Supplementary material for: ASCL1 regulates and cooperates with FOXA2 to drive terminal neuroendocrine phenotype in prostate cancer
Source: JCI Insight. 2024 Dec 6;9(23):e185952. doi: 10.1172/jci.insight.185952 (PMC11623946; doi:10.1172/jci.insight.185952)
Supplement: Supplemental data [file jciinsight-9-185952-s079.pdf]

Figure S1. ASCL1 regulates FOXA2 in terminal NEPC

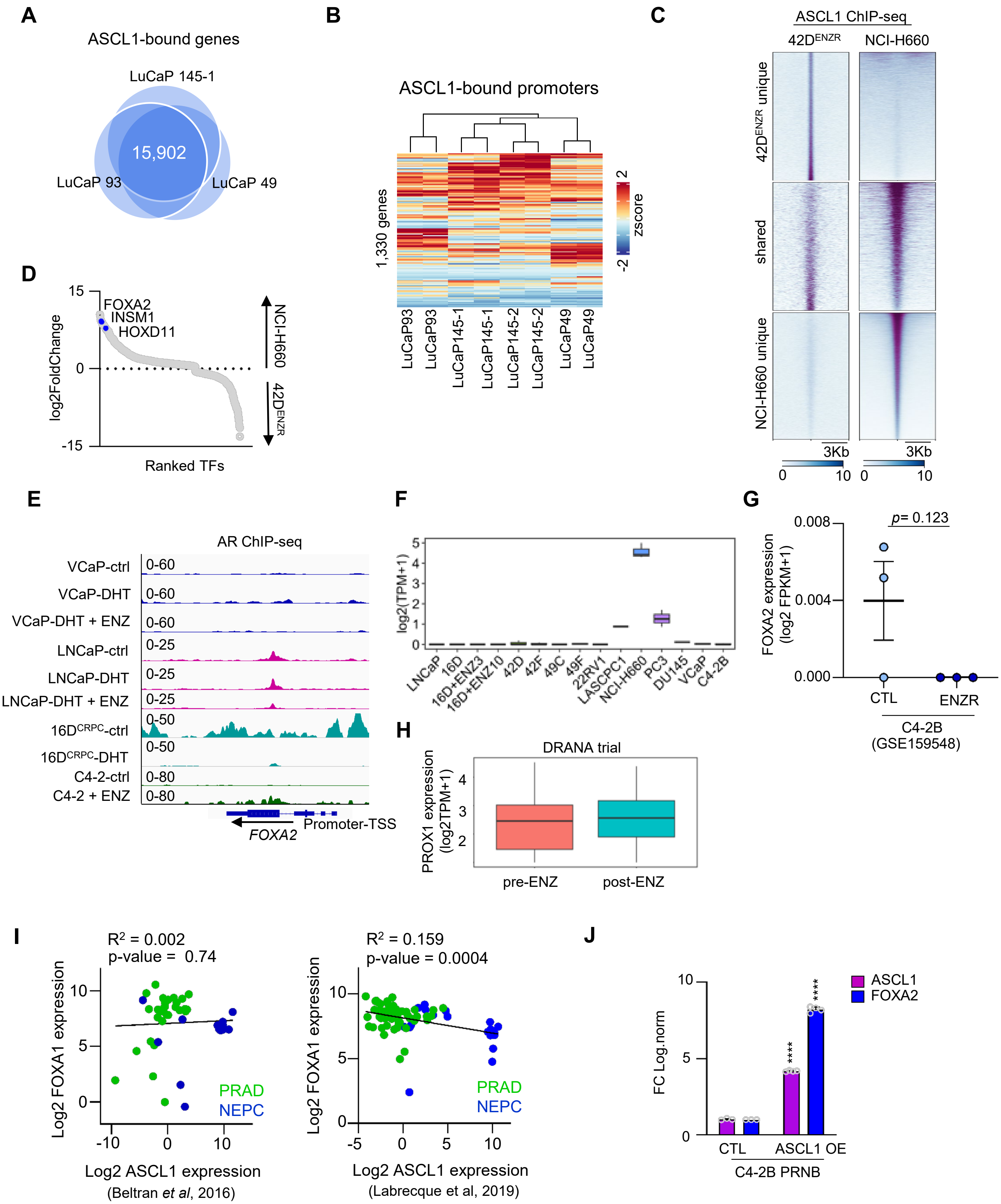

Figure S1. ASCL1 regulates FOXA2 in terminal NEPC

A. Genes co-bound by ASCL1 in three LuCaP PDXs.
B. Heatmap shows expression of all (1,330) ASCL1-bound promoters in ASCL1-driven LuCaP patient-derived xenografts. Data are represented as z-score.
C. Heatmap of unique ASCL1 binding intensity in tNEPC cell line $42D^{ENZR}$ and de novo NEPC cell line NCI-H660. Data is presented as fold change over input, with each horizontal line representing a 3 kb locus.
D. Transcription factors (TFs) expression enriched in de novo NEPC cell line NCI-H660 compared to tNEPC cell line $42D^{ENZR}$. TFs are ranked based on log2 fold change.
E. AR binding at FOXA2 promoter region shown using IGV tracks in a panel of prostate cancer cell lines treated with DHT or ENZ.
F. Expression of FOXA2 shown as log2 TPM+1 in a panel of prostate cancer cell lines as well as in CRPC ($16D^{CRPC}$) following 3 and 10 days of enzalutamide (ENZ) treatment.
G. Expression of FOXA2 shown as log2 FPKM+1 in C4-2B CTL and after ENZ treatment.
H. Expression of FOXA2 shown as log2 TPM+1 in the patient dataset (DRANA trial) (41) pre- and post-enzalutamide treatment.
I. Correlation between ASCL1 and FOXA1 expression in patient datasets (Beltran et al) (9) (left), and (Labrecque et al) (3) (right).
J. Expression of ASCL1 and FOXA2 in C4-2B PRNB following overexpression ASCL1 (ASCL1OE) shown as fold change of logged normalized count (FC log.norm). Data are reported as mean ± SD; $p < 0.05$ (CTL n = 4 and ASCL1 OE n = 4 biologically independent samples) (ASCL1 and FOXA2 $p < 0.00001$).

Figure S2. FOXA2 in NEPC and SCLC

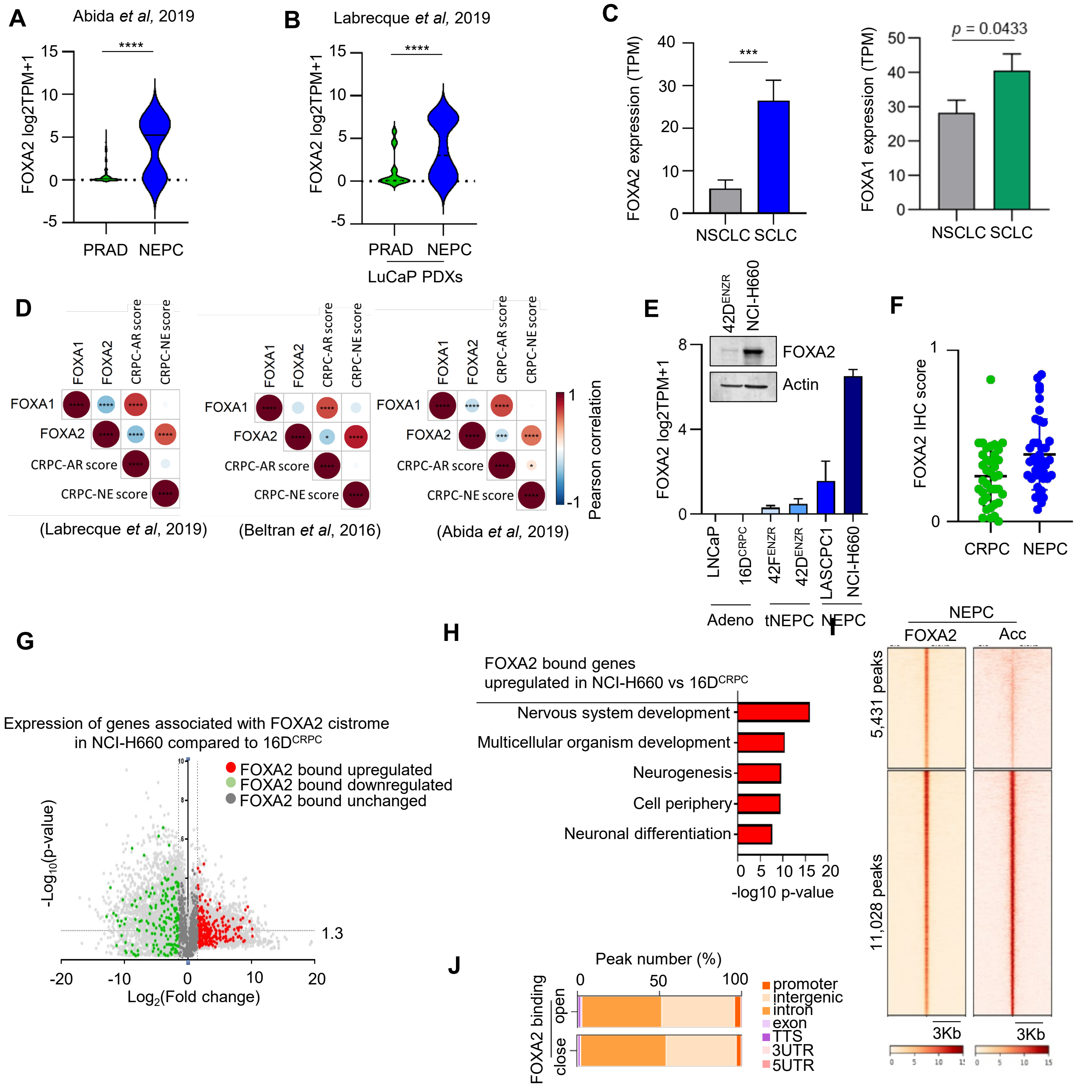

Figure S2. ASCL1 regulates FOXA2 in terminal NEPC

A. FOXA2 gene expression (log2TPM+1) in patient datasets (46). Violin plots show median (middle solid line), quartiles as dotted lines and interquartile range, with significance assessed using a two-tailed unpaired t-test.

B. FOXA2 gene expression (log2TPM+1) in LuCaP PDXs (3). Violin plots show median (middle solid line), quartiles as dotted lines and interquartile range, with significance assessed using a two-tailed unpaired t-test.

C. FOXA1 and FOXA2 mRNA expression (TPM) in small cell (SCLC) and non-small-cell lung cancers (NSCLC) cell lines (NSCLC n = 71 and SCLC n = 50) (47). Each dot represents an individual patient. Data is shown as mean ± SD, with significance assessed using a two-tailed unpaired t-test. ($*p < 0.05$, $**p < 0.001$, $***p < 0.0001$).

D. The correlation between FOXA1 and FOXA2 mRNA expression with AR or NE score (9) in patient datasets (3, 9, 61) using Pearson correlation.

E. FOXA2 gene expression (log2TPM+1) in Adeno (LNCaP and $16D^{CRPC}$), tNEPC ($42D^{ENZR}$ and $42F^{ENZR}$), and NEPC (LASCPC1 and NCI-H660) cell lines.

F. FOXA2 staining intensity was quantified, mean ± SD, CRPC (n = 40) and NEPC (n = 26); two-tailed unpaired t-test (p-value = 0.0058).

G. Volcano plot shows differentially regulated FOXA2 bound genes in NCI-H660 compared to $16D^{CRPC}$. Red shows up-regulated (log2 fold change >1), green shows down-regulated (log2 fold change <-1), and gray shows unchanged genes.

H. Pathways associated with upregulated FOXA2 bound genes in NCI-H660 compared to $16D^{CRPC}$ were identified using gProfiler. $p<0.05$.

I. Heatmap represents the FOXA2 ChIP-seq binding intensity and chromatin accessibility in the NCI-H660 cell line. Each horizontal line represents a 3-kb locus.

J. Genomic annotation is shown as a percentage of all peaks showing FOXA2 binding in open or close chromatin.

Figure S3. FOXA2 cooperates with ASCL1 in small-cell cancers

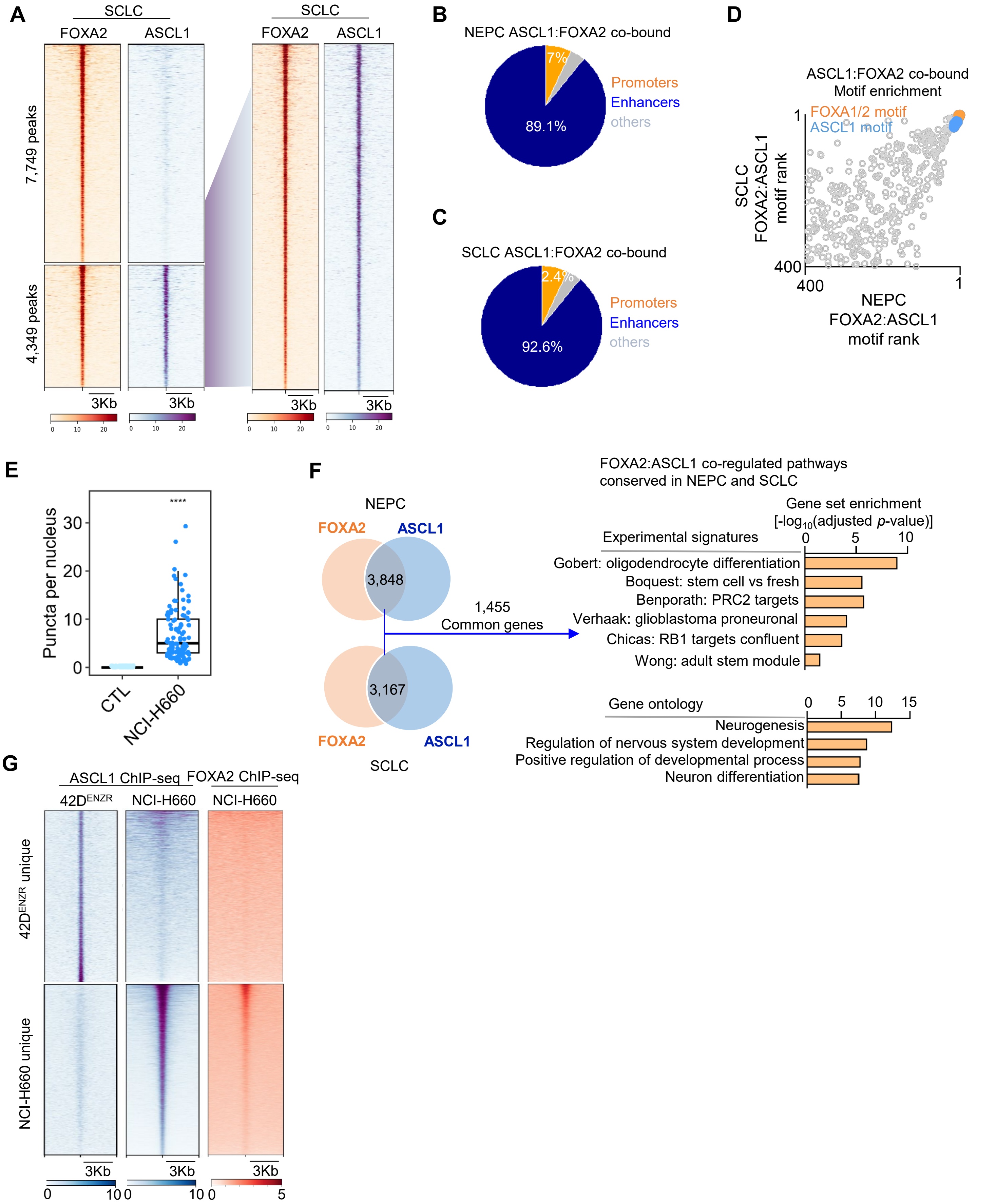

Figure S3. FOXA2 cooperates with ASCL1 in small-cell lung cancer

A. Heatmap of FOXA2 and ASCL1 ChIP-seq binding intensity in NCI-H889 cells. Each horizontal line represents a 3-kb locus. The heatmap represents the regions co-occupied by FOXA2 alone or FOXA2 and ASCL1.
B. Genomic annotation of ASCL1:FOXA2 co-bound regions in NEPC presented as a percentage of total peaks. Intron and intergenic regions were called enhancers.
C. Genomic annotation of ASCL1:FOXA2 co-bound regions in SCLC presented as a percentage of total peaks. Intron and intergenic regions were called enhancers.
D. TF binding motif enrichment surrounding ASCL1:FOXA2 co-bound regions in SCLC and NEPC. Motifs were ranked based on differential p-value. Each dot represents a motif. Orange shows the FOXA motif family (FOXA1 and FOXA2), and blue shows the ASCL1 motif.
E. PLA quantification showing ASCL1 and FOXA2 interaction as puncta per nuclei in control (CTL) and NCI-H660 cell line.
F. Genes co-bound by ASCL1 and FOXA2 in NEPC and SCLC. Pathway analysis was performed using gProfiler on common genes between SCLC and NEPC. $p<0.5$.
G. Heatmap of unique ASCL1 binding intensity overlayed with FOXA2 ChIP-seq in regions unique to $42D^{ENZR}$ and NCI-H660 cell lines. Data is presented as fold change over input, with each horizontal line representing a 3 kb locus.

Figure S4. ASCL1 and FOXA2 co-regulate PROX1

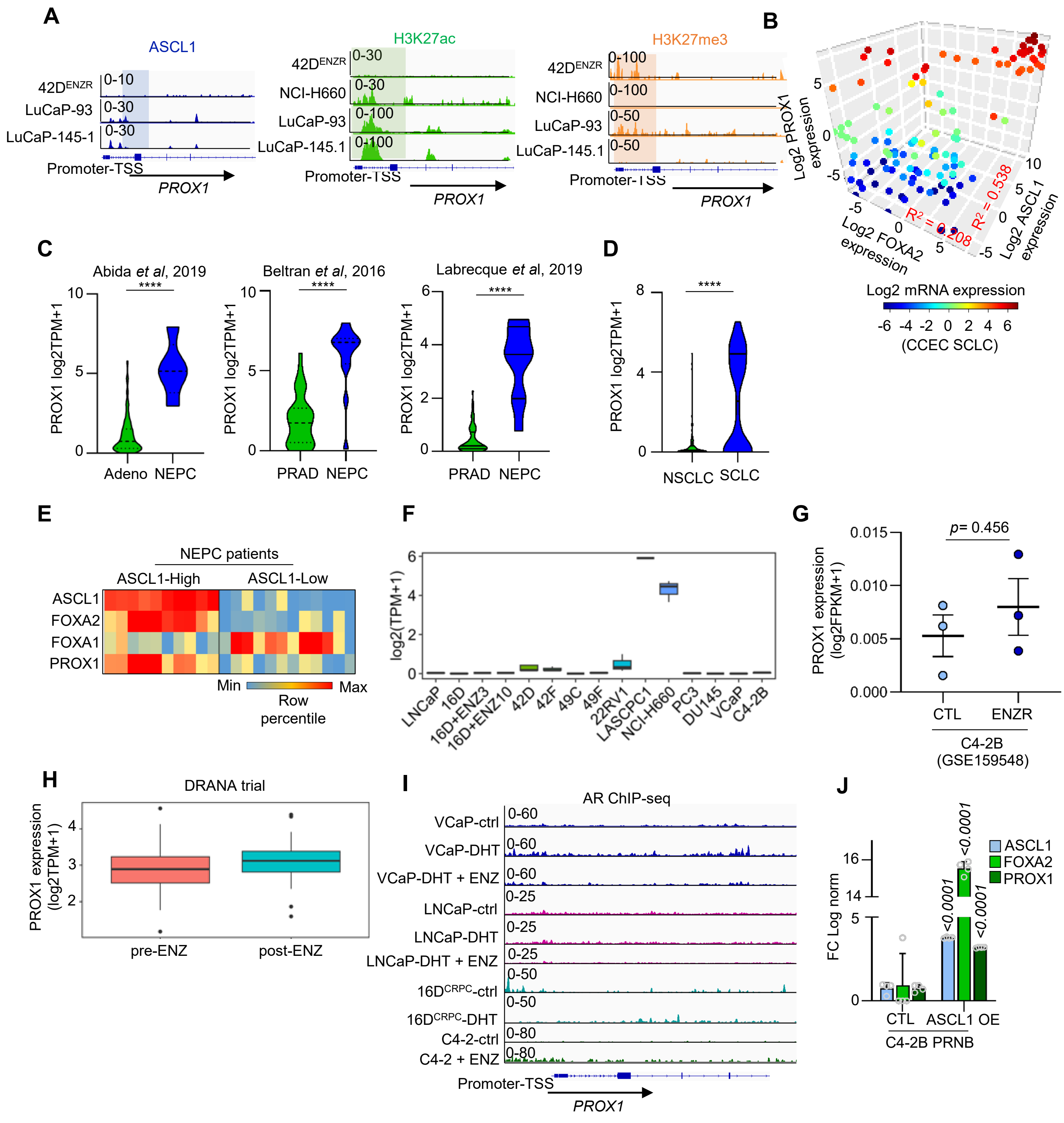

Figure S4. ASCL1 and FOXA2 co-regulate PROX1

A. IGV tracks show ASCL1 binding (left), H3K27ac signal (middle), and H3K27me3 signal (right) on PROX promoter in $42D^{ENZR}$, NCI-H660, and LuCaPs 93 and 145-1.
B. The correlation between PROX1 mRNA expression with FOXA2 and ASCL1 in the SCLC dataset (47), with each dot representing a patient tumor and the colour reflecting PROX1 mRNA expression (log2 TPM); p-value < 0.05, with significance assessed by two-tailed unpaired t-test.
C. PROX1 expression presented as log2TPM in prostate cancer patient datasets (3, 9, 61).
D. PROX1 expression presented as log2TPM in lung cancer datasets (47).
E. Heatmap shows the expression of ASCL1, FOXA1/2, and PROX1 in Labrecque et al dataset (3), presented as log2TPM with each row showing the minimum and maximum row percentile.
F. Expression of PROX1 shown as log2 TPM+1 in a panel of prostate cancer cell lines as well as in CRPC ($16D^{CRPC}$) following 3 and 10 days of enzalutamide (ENZ) treatment.
G. Expression of PROX1 shown as log2 FPKM+1 in C4-2B CTL and after ENZ treatment.
H. Expression of PROX1 shown as log2 TPM+1 in the patient dataset (DRANA trial) (41) pre- and post-enzalutamide treatment.
I. AR binding at PROX1 promoter region shown using IGV tracks in a panel of prostate cancer cell lines treated with DHT or ENZ.
J. ASCL1, FOXA2 and PROX1 gene expression in C4-2B PRNB cell line (CTL) and following overexpression of ASCL1 (ASCL1 OE). Data are reported as fold change of log2 normalized count, and significance is assessed using a two-tailed unpaired t-test (CTL and ASCL1 OE n = 3 biologically independent samples).

Figure S5. PROX1 is required for NE phenotype characteristics and proliferation

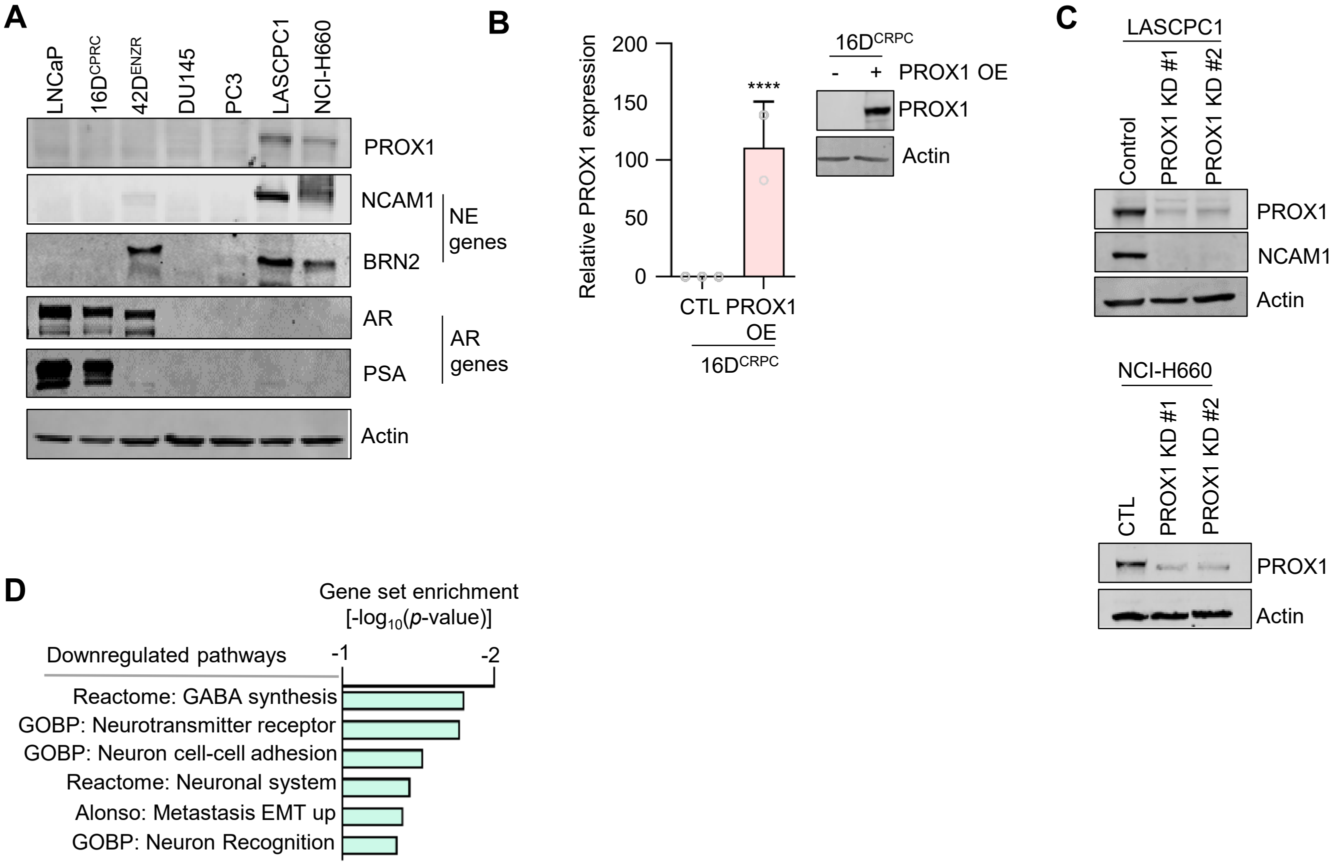

Figure S5. PROX1 is required for NE phenotype characteristics and proliferation

A) Western blot shows the expression of PROX1, NE markers (BRN2 and NCAM1), AR and AR marker (PSA) in a panel of prostate cancer cell lines, with actin used as a loading control.

B) PROX1 expression shown as relative mRNA (left) and protein expression (right) in $16D^{CRPC}$ following PROX1 overexpression (OE). mRNA data was normalized to GAPDH. Actin is used as a loading control.

C) Western blot shows the expression of PROX1 and NE marker NCAM1 in the LASCPC1 cell line (top) and NCI-H660 cell line (bottom) following knockdown of PROX1 using two different shRNA.

D) Pathways downregulated in LASCPC1 following knockdown of PROX1 (shPROX1) shown as –log10 p-value with $p<0.05$.
