# Supplementary figures and images for: ASCL1 regulates and cooperates with FOXA2 to drive terminal neuroendocrine phenotype in prostate cancer
Source: JCI Insight. 2024 Dec 6;9(23):e185952. doi: 10.1172/jci.insight.185952 (PMC11623946; doi:10.1172/jci.insight.185952)

Figure 3C

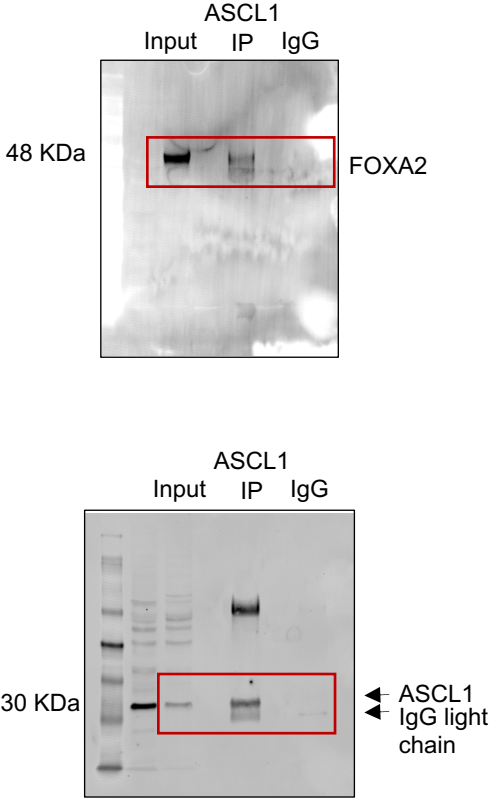

Figure S2E

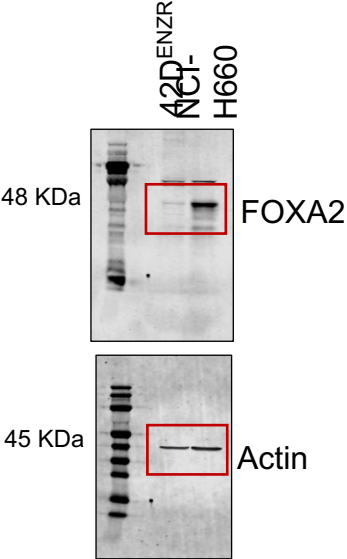

Figure S5B

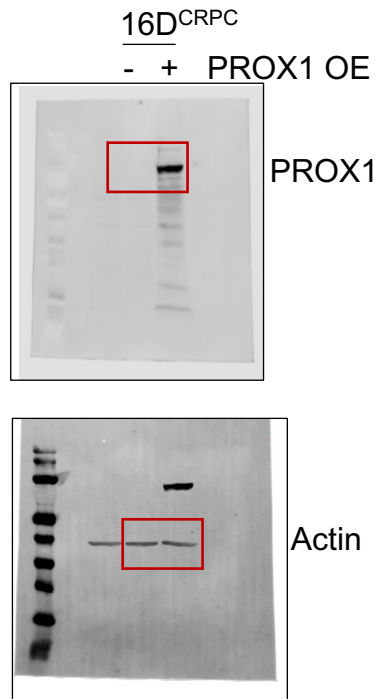

Figure S5C

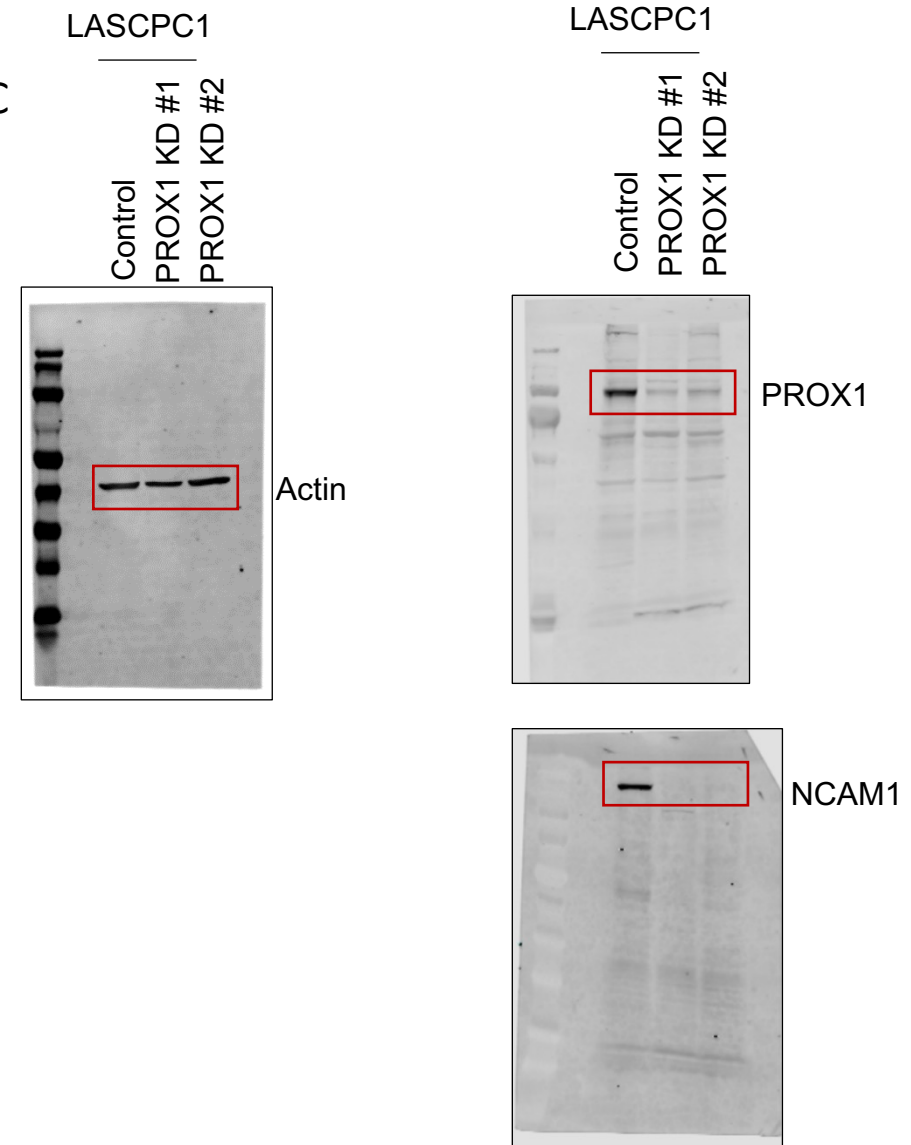

Figure S5A

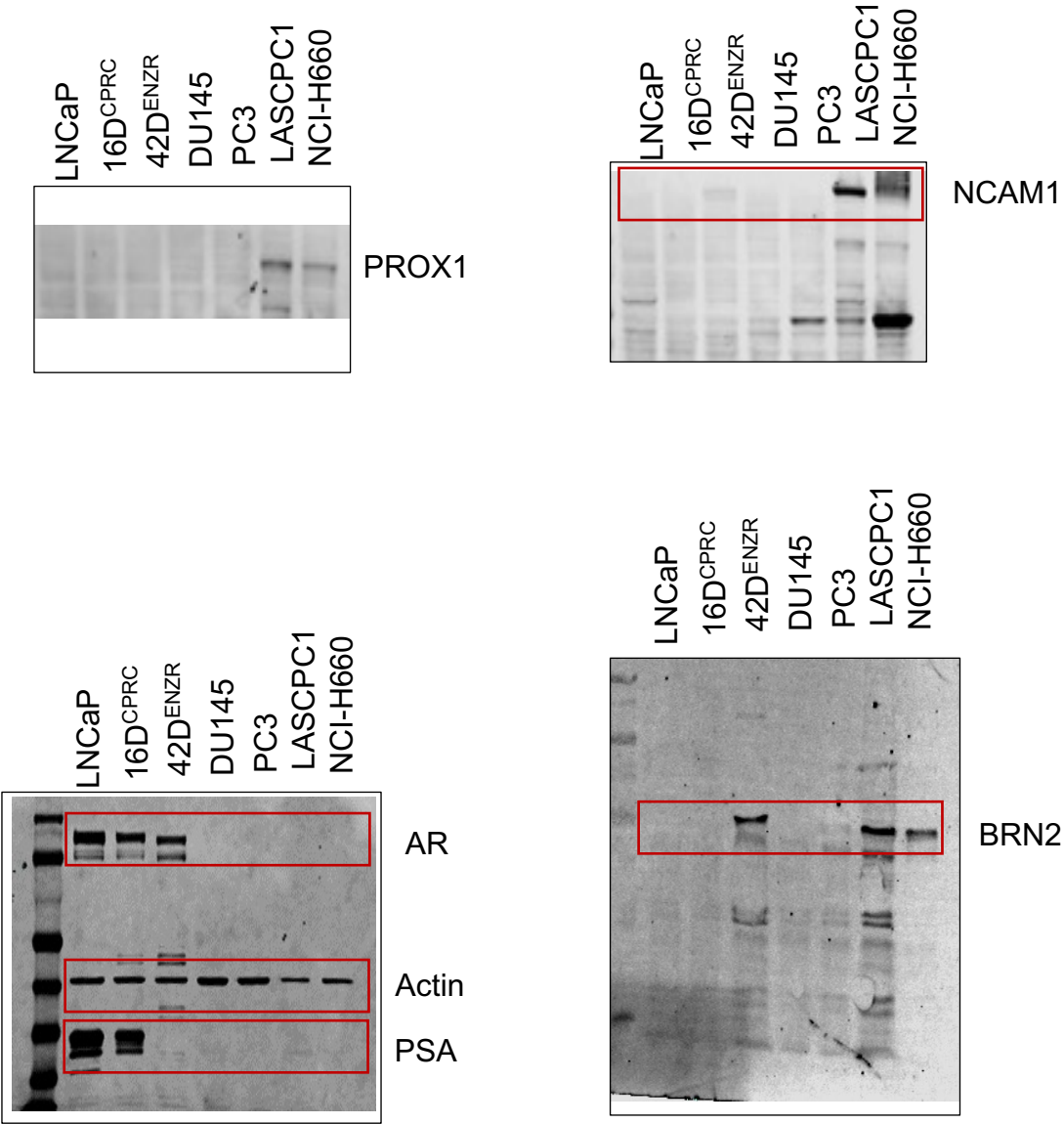

Supplement: Unedited blot and gel images [file jciinsight-9-185952-s080.pdf]
